# Mycotoxins’ Toxicological Mechanisms Involving Humans, Livestock and Their Associated Health Concerns: A Review

**DOI:** 10.3390/toxins14030167

**Published:** 2022-02-24

**Authors:** Chinaza Godseill Awuchi, Erick Nyakundi Ondari, Sarah Nwozo, Grace Akinyi Odongo, Ifie Josiah Eseoghene, Hannington Twinomuhwezi, Chukwuka U. Ogbonna, Anjani K. Upadhyay, Ademiku O. Adeleye, Charles Odilichukwu R. Okpala

**Affiliations:** 1 Department of Biochemistry, Kampala International University, Bushenyi P.O. Box 20000, Uganda; drerickondarin@gmail.com (E.N.O.); sonwozo@yahoo.com (S.N.); grace.odongo@kiu.ac.ug (G.A.O.); ifiejosiah.ese@kiu.ac.ug (I.J.E.); 2 Department of Chemistry, Kyambogo University, Kyambogo, Kampala P.O. Box 1, Uganda; thannington@yahoo.com; 3 Department of Biochemistry, Federal University of Agriculture, P.M.B. 2240, Abeokuta 110124, Ogun State, Nigeria; ogbonnacu@funaab.edu.ng; 4 Heredity Healthcare & Lifesciences, 206-KIIT TBI, Patia, Bhubaneswar 751024, Odisha, India; upadhyayanjanikumar6@gmail.com; 5 Faith Heroic Generation, No. 36 Temidire Street, Azure 340251, Ondo State, Nigeria; adeleyedsegun@gmail.com; 6 Department of Functional Foods Product Development, Wrocław University of Environmental and Life Sciences, 51-630 Wrocław, Poland

**Keywords:** mycotoxins, mycotoxicosis, molds, disease, health risks

## Abstract

Mycotoxins are well established toxic metabolic entities produced when fungi invade agricultural/farm produce, and this happens especially when the conditions are favourable. Exposure to mycotoxins can directly take place via the consumption of infected foods and feeds; humans can also be indirectly exposed from consuming animals fed with infected feeds. Among the hundreds of mycotoxins known to humans, around a handful have drawn the most concern because of their occurrence in food and severe effects on human health. The increasing public health importance of mycotoxins across human and livestock environments mandates the continued review of the relevant literature, especially with regard to understanding their toxicological mechanisms. In particular, our analysis of recently conducted reviews showed that the toxicological mechanisms of mycotoxins deserve additional attention to help provide enhanced understanding regarding this subject matter. For this reason, this current work reviewed the mycotoxins’ toxicological mechanisms involving humans, livestock, and their associated health concerns. In particular, we have deepened our understanding about how the mycotoxins’ toxicological mechanisms impact on the human cellular genome. Along with the significance of mycotoxin toxicities and their toxicological mechanisms, there are associated health concerns arising from exposures to these toxins, including DNA damage, kidney damage, DNA/RNA mutations, growth impairment in children, gene modifications, and immune impairment. More needs to be done to enhance the understanding regards the mechanisms underscoring the environmental implications of mycotoxins, which can be actualized via risk assessment studies into the conditions/factors facilitating mycotoxins’ toxicities.

## 1. Introduction

Mycotoxins are toxic metabolic compounds produced by some molds. Many mycotoxins have chemical stability and can survive the rigors encountered throughout the food supply chain. The most common mycotoxins of concern to humans and livestock include aflatoxins, citrinin, ochratoxins, fumonisins, patulin, zearalenone, nivalenol, deoxynivalenol, fumonisins, and ergot alkaloids. The production of some mycotoxins occurs mainly in the field, while, for others, it can happen both in the field and in the postharvest period. Health effects involving mycotoxins in humans and animals include specific diseases or health issues, a weak immune system with no specificity to a particular mycotoxin, death, and action as irritants or allergens. Certain mycotoxins have been found to be harmful to most other microorganisms [1,2,3]. Some mycotoxins, such as aflatoxins and fumonisins, interfere with protein synthesis, cause cancer, prevent particle clearance of the lungs, damage macrophage systems, and raise sensitivity to bacterial endotoxins [2,3]. In the food and feed industries, there has been an increase in the usage of mycotoxin-binding agents such as bentonite clays or montmorillonite for effective adsorption and removal of mycotoxins. However, not all mycotoxins bind to such mycotoxin-binding agents. Another approach to control mycotoxin involves its deactivation. With yeast (*Trichosporon mycotoxinvorans*), enzymes (esterase), or bacterial strains (*Eubacterium* BBSH 797), mycotoxin levels can be drastically lessened before harvesting [3,4]. Some methods of mycotoxin removal make use of physical separation, nixtamalization, heat treatment, washing, cleaning, milling, radiation, biological or chemical agents, and extraction with solvents [5]. In particular, the irradiation method has been shown to have high efficacy against the growth of mold and the presence of mycotoxins [3,4].

Mycotoxins of major toxic interest include aflatoxins, deoxynivalenol, fumonisins, ochratoxin A, and citrinin, partly due to their increased frequency and high occurrence in foods and feeds commonly consumed by humans and animals. The toxicities of aflatoxins, fumonisins, deoxynivalenol, and ochratoxin A include cytotoxicity, liver cancer, kidney cancer and damage, intestinal barrier function disruption, immune modulation, and poor fetal development, all of which can affect humans. In general mycotoxins pose challenges to humans and animals worldwide due to their recently increasing occurrence and their toxicities. Mycotoxins enter the food chain due to mold infestation of crops. Mycotoxin exposure can take place directly via the consumption of infected foods and feeds or indirectly through livestock given infected feeds, particularly from milk and dairy products [2]. The health effects of certain food-borne mycotoxins have been reported to be acute, whereby the symptoms of severe sickness appear readily following the ingestion of foods polluted with the mycotoxin. Some mold metabolites in foods have prolonged health effects on human and animal, including immune deficiency and the induction of cancers [2]. Among the hundreds of mycotoxins known to humans, around a handful have drawn the most concern because of their occurrence in food and severe effects on human health. For instance, the *Fusarium* mycotoxins are made by at least 50 *Fusarium* species, and they pollute the grains of the growing cereals including wheat, maize, and millet [6,7,8]. Most fungi thrive well in oxygen and in very little quantities given the diminutive sizes of their spores. The fungi/molds consume organic materials wherever the environmental and surrounding conditions are suitable, forming colonies and raising the amount of released mycotoxins. The actual motivation for mycotoxin release is yet unknown, as it is not required to grow or develop the fungi [9].

A summary of recently conducted reviews on mycotoxin toxicology involving humans and livestock, as well as control/removal strategies, is presented in Table 1. Some researchers introduced the natural occurrence of *Alternaria* mycotoxins, as well as their toxicity, metabolism, and analytical methods [10], whereas others discussed the co-occurrence of masked mycotoxins, as well as their sampling and extraction, and the suitability of LC–MS/MS for accurate and precise analysis/detection [11]. Additionally, the occurrence of mycotoxins, their toxic effects, the detoxifying agents, their qualitative and quantitative analysis (for modified mycotoxins), and the most important mycotoxins in crops/finished fish feed have been reported [12,13,14,15,16,17]. Consumer health safety concerns, mitigation/treatment strategies associated with mycotoxin toxicities, and how they affect animals, foods, humans, and plants remain very crucial [5,18]. Clearly, the body of knowledge on this subject matter is continually growing. Our analysis of recently conducted reviews showed that toxicological mechanisms associated with mycotoxins deserve additional attention. Understanding the toxicological mechanisms associated with mycotoxins is crucial given the fact that mycotoxins make the host weak, thereby providing the fungi with an increased chance to thrive further and cause more harm. Accordingly, it is clear that the making and the release of mycotoxins depend on the intrinsic and extrinsic environmental conditions. Thus, the metabolites vary greatly, particularly in terms of toxic potency, which to a large extent depends on the infected host and its vulnerability, defense mechanisms, and metabolism [19]. Although mycotoxins have been known for years, they are also recognized as emerging contaminants, largely due to new discoveries/knowledge [18,20]. Given the increasing global public health importance of mycotoxins, there is a need for continued review of the relevant literature, to expand understanding especially as it relates to their toxicological mechanisms. To supplement existing knowledge, therefore, this current work reviewed mycotoxins’ toxicological mechanisms involving human, livestock, and their associated health concerns. A succinct discourse on some mycotoxins directly involved in some types of cancer, as well as mycotoxins’ actions on the human cellular genome, is included.

## 2. Mycotoxins and Mycotoxicosis

Mycotoxicosis is when exposure to mold substances/mycotoxins brings about poisoning. Mycotoxicosis can cause acute and chronic health effects to humans and livestock via ingestion, inhalation, and contact with the skin, as well as through entering the lymphatic system and blood stream. While acute effects manifest within 72 h of exposure, chronic effects take more than 72 h and may run into months, years, or even decades. The symptoms and effects of mycotoxicosis depend on the type of mycotoxin, although two or more mycotoxins may have similar effects [3]. Generally, most health effects of mycotoxins in humans and animals in toxic doses include identifiable diseases, weak immunity without any trace to one toxin, identifiable health problems, death, and action as allergens or irritants. A number of mycotoxins are destructive to other microbes, e.g., fungi or bacteria [22]. Mycotoxins in stored animal feed have been suggested to be the cause of the rare phenotypical sex changes in hens, causing them to resemble and act as if they were male [23]. Mycotoxins have the potential for chronic and acute health effects [2,24], via inhalation and entry into the lymphatic system and blood stream. They harm the macrophage system, impair protein synthesis, intensify the response to bacterial endotoxin, and inhibit particle clearance of the lungs [25]. Symptoms of mycotoxicosis are based on the type of mycotoxin, the age, sex, and health of victims, the mycotoxin concentration, and the length of exposure [4,25]. The synergistic effects connected with many factors, e.g., diet, genetic makeup, and the relations with different toxins, have not been studied sufficiently. Consequently, there is a likelihood that the vitamin deficiencies, alcohol abuse, caloric deprivation, and infectious diseases can compound mycotoxicosis [2,25]. Mycotoxin infestation of medicinal plants and other plant products can increase health issues in human, thus symbolizing a special concern [26,27]. Natural occurrences of the mold toxins in herbal medicines and medicinal plants have been reported in countries such as Spain, India, Turkey, China, and Germany, as well as regions such as the Middle East [26,27]. 

In the 1990s, there were public health fears over the increased mycotoxin occurrence, which brought about millions of dollars’ worth of mold settlements. This was a direct result of a study conducted by the US Center for Disease Control in Cleveland, Ohio, which provided evidence with regard to the connection between mycotoxins in pulmonary hemorrhage in infants and the spores of *Stachybotrys* [28]. However, on the basis of internal and external data reviews in 2000, the Center for Disease Control (CDC) resolved that, due to pitfalls in some of their methodologies, the connection was not proven. The spores of *Stachybotrys* in studies involving animal models have been reported to result in lung hemorrhaging, but only when the concentration is too high. The Center of Integrative Toxicology, Michigan State University, carried out a study which examined the cause of damp building-related illness (DBRI), identifying *Stachybotrys* as a possible contributing factor. So far, studies on animals have shown that airway exposure to *Stachybotrys chartarum* can cause allergy, cytotoxicity, and inflammation in the lower and upper respiratory tracts. Trichothecene mycotoxicosis seems to be a factor in the fundamental cause of a number of these health effects. Findings have shown that lower doses may even cause the same symptoms [29]. A great number of toxicologists utilize the concentration of no toxicological concern (CoNTC) to describe the airborne concentrations of mycotoxin, which are strongly believed not to cause harm to humans following continuous exposure across a 70 year lifetime. Dimorphic fungi, e.g., *Paracoccidioides brasiliensis* and *Blastomyces dermatitidis*, are believed to cause an endemic form of systemic mycoses. Between 2005 and 2011, there was an outbreak of dog foods contaminated with aflatoxins. The residents of affected areas became concerned about the effects of mycotoxins. Mycotoxins in fodders, including in silage, have the potential to reduce the performance of farm animals and may even lead to their death. Upon being eaten by cattle, many mycotoxins lessen the yield of milk. Additionally, the release of mycotoxins in food crops affects the nutritional composition of the foods and feeds [30].

In a study involving plant-based dietary (nutritional) supplements in 2015, the peak concentration of mycotoxins was reported to be around 37 mg per kg specific to milk thistle-based supplements [31]. Mycotoxins resist breakdown or decomposition during digestion, and they remain in the meat and milk product food chain. Temperature processing, such as freezing and cooking, may not necessarily deter all mycotoxins [32]. For instance, in the food and feed industries, there is a common practice to remove mycotoxins by adding binding agents, e.g., bentonite clay or montmorillonite [33], which are aimed at removing or at least reducing the number of mycotoxins that withstand other preprocessing measures. To reverse the adverse effects of mycotoxins, the criteria adopted to examine functionality include (a) a low effective inclusion rate, (b) the affirmation of interaction between the adsorbent and mycotoxin, (c) the efficiency of the active substance verified by scientific data with evidence, (d) a high affinity to absorb mycotoxins at low concentration, (e) a high ability to absorb a high concentration of mycotoxins, (f) environmentally friendly and nontoxic substances, (g) proven data with all major mycotoxins in vivo, and (h) stability over different pH levels [3,4,33]. As most mycotoxins withstand the rigors of food and feed processing, current tactics for controlling mycotoxins have aimed at their deactivation. With yeast (*Trichosporon mycotoxinvorans*), enzymes (esterase, de-epoxidase), or bacterial strains (*Eubacterium* BBSH 797), mycotoxin levels can be drastically reduced before harvesting. Other methods of mycotoxin removal make use of physical separation, nixtamalization, heat treatment, washing, cleaning, milling, radiation, biological or chemical agents, and extraction with solvents. In particular, the irradiation method has been shown to have high efficacy against the growth of mold and mycotoxins [3,4,33].

## 3. Mycotoxins: Their Toxicological Mechanisms and Associated Health Concerns

Fungal infestation of agricultural crops facilitates the entry of mycotoxins into the food chain. This can be via animal feeds or direct consumption by man. In 2004, 125 individuals lost their lives in Kenya and around 200 received treatment after consuming aflatoxin-infested maize [28,34,35]. Their demise was mostly connected with domestically cultivated maize which was not properly dried before storage or treated with fungicides. Due to food insecurity at that period, farmers might have harvested maize immaturely to circumvent burglaries from their farmyards, resulting in the grain not maturing fully and being more vulnerable to infection by mold. Other common substrates susceptible to mycotoxigenic fungal growth and mycotoxin release include cereals, nuts, spices (e.g., red chili, dry ginger, and black pepper), and dried fruit [36]. Many mycotoxins have been shown to exhibit toxicities in several ways. The toxic nature of mycotoxins can lead to various fatal diseases in animal and human environments, due to the harmful biochemical substances released by the molds that are able to readily colonize agricultural crops [1,3]. Fungal growths can occur at any time, e.g., on farms, during or after harvest, in storage facilities, and in foods that are usually stored in warm, humid, or damp environmental conditions [2,4]. *Fusarium* toxins include various mycotoxins, such as trichothecenes, which are mostly connected with fatal and chronic harmful effects in humans and animals, zearalenone, which has not been associated with any fatality in humans or animals, and fumonisins, which affect CNS of horses and might induce cancer in rodents. Other *Fusarium* mycotoxins include beauvercin, equisetin, butenolide, and enniatins [37].

Moreover, many if not all mycotoxins have a reasonable level of chemical stability, which enables them to survive the rigors of food processing. Generally, it is widely accepted that there are hundreds of different mycotoxins in nature. However, the most commonly known mycotoxins that are specifically of concern to humans and livestock include aflatoxin, citrinin, ochratoxin, patulin, trichothecenes, zearalenone, nivalenol/deoxynivalenol, fumonisins, and ergot alkaloids such as ergotamine [2,25,38]. In general, a single mold species can make several mycotoxins, and many species of mold might release the same mycotoxins. Mycotoxin types, described along with the foods in which they are mostly found and their respective toxicities, are articulated in Table 2. Indeed, the toxicities of these mycotoxins raise various health concerns, with carcinogenic, mutagenic, hepatotoxic, nephrotoxic, genotoxic, and/or biotoxicological elements. Indeed, the mycotoxins covered here include aflatoxins (aflatoxins B1, B2, G1, G2, M1, M2) [2,39], ochratoxin A [40,41], deoxynivalenol (DON) [42], fumonisins (fumonisins B1, B2, B3, B4) [43,44,45,46], zearalenone (ZEA), also known as F-2 mycotoxin [3,47], patulin, citrinin [38,42], ergot alkaloids [25], and T-2, which is a trichothecene mycotoxin [48,49,50]. Other mycotoxins captured in Table 2 include diacetoxyscirpenol (DAS) or 4,15-diacetoxyscirpenol (DAS), also referred to as anguidine [51,52,53], fusarenon X (FusX) [54,55,56], and nivalenol (NIV) [57,58]. In subsequent sections, we discuss these mycotoxins in greater detail with respect to their mechanisms of action, as well as the ailments they cause.

### 3.1. Aflatoxins

Aflatoxins are produced by several species of *Aspergillus,* especially *Aspergillus parasiticus* and *Aspergillus flavus*, in many commodities [59]. Aflatoxin generally involves four types: aflatoxins B1, B2, G1, and G2 (AFB1, AFB2, AFG1, and AFG2, respectively). Together, all aflatoxins are generally referred to as total aflatoxin. They are mostly connected with the agricultural commodities farmed in tropical and subtropical regions, e.g., peanuts (groundnuts), spices, cotton, maize, and pistachios [59,60]. Maize and groundnuts are the most affected commodities in tropical regions such as sub-Saharan Africa, although many agricultural commodities can be affected. Aflatoxins are well-known toxic mycotoxins released by some molds that grow in hay, grains, decaying vegetation, and soil. Crops frequently affected by such molds include cereals (e.g., wheat, sorghum, rice, acha, millet, guinea corn, and corn), tree nuts (e.g., almond, pistachio, coconut, and walnut), oilseeds (e.g., peanut, sunflower, cotton seeds, soybean, and sesame), and spices (e.g., garlic, black pepper, coriander, turmeric, ginger, and chili peppers). Aflatoxin B1 (AFB1) is the most toxic and a strong carcinogenic toxin directly linked to many health problems, including liver cancer, in several animals [2,28,59]. Such mycotoxins can also be seen in animal milk and dairy products, especially animals fed with infected feeds, such as aflatoxin M1 (AFM_1_) [2]. AFM1 is a product of AFB1 detoxication and is commonly found in milk and dairy products. The main sources of aflatoxins in feeds are peanut, meal, maize, and cottonseed meal. The World Health Organization stated that large doses of aflatoxins can result in acute poisoning, known as aflatoxicosis, which can be life-threatening, often through liver damage; aflatoxins have also been reported to be genotoxic, which means that they can harm DNA and cause cancer in animals. There is sufficient evidence to show that aflatoxins cause hepatic (liver) cancer in animals and humans.

#### 3.1.1. Mechanisms of Action of Aflatoxins

With a focus on the carcinogenicity and mutagenicity of aflatoxins, several studies have been carried out on aflatoxin B1, which, because of the double bond at position 8, 9, is usually metabolized to AFB1-8,9-epoxide (its reactive form), and this can bind to cellular macromolecules such as deoxyribonucleic acid (DNA) [61,62,63]. AFB-N7-guanine, a pro-mutagenic lesion, is the main DNA adduct, and it commonly leads to G→T transversions. AFB-N7-guanine is detected in urine, where it serves as a biomarker for exposure in epidemiological research. In general, AFG2 and AFB2 are less biologically active because they do not have the 8,9 double bond. Aflatoxin G1 can be biologically activated to 8,9-epoxide; however, it is less mutagenic compared to aflatoxin B1, showing the respective epoxides’ stearic chemistry; AFB1-8,9-epoxide readily intercalates into the double helix of DNA compared to aflatoxin G1, leading to the formation of higher DNA adduct levels for any specific dose [28,60,64]. Aflatoxin M1, unlike AFM2, has the 8,9 double bond; consequently, AFM1 can be biologically activated to 8,9-epoxide, which is reactive.

**Figure 1:**
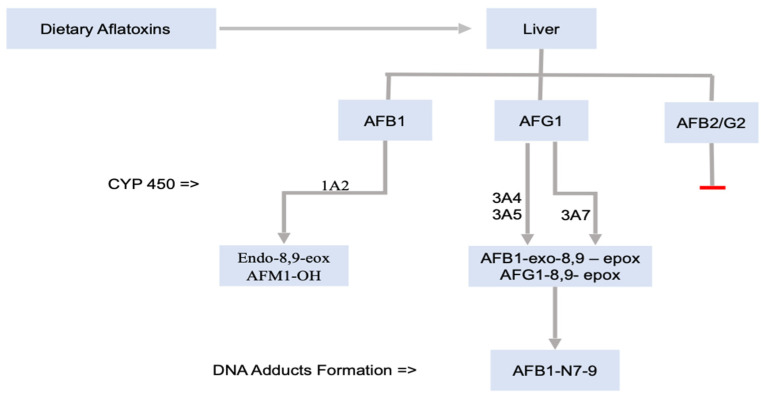
The metabolism of aflatoxins in the liver. AFB1 = aflatoxin B1; AFB2 = aflatoxin B2; AFG1 = aflatoxin G1; AFG2 = aflatoxin G2; CYP450 = cytochrome P450; 1A2 = CYP1A2 = cytochrome P450 1A2; 3A4 = CYP3A4 = cytochrome P450 3A4; 3A5 = CYP3A5 = cytochrome P450 3A5; 3A7 = CYP3A7 = cytochrome P450 3A7. Red line signals the mechanistic pathway of AFB2/G2 do not differ much from AFB1/G1.

Figure 1 shows how aflatoxin exposure through the diet moves in the liver, producing different toxicities. The main cytochrome P450 (CYP450) enzymes in human involved in the metabolism of aflatoxins are CYP3A5, CYP1A2, and CYP3A4, and the liver is the major site of bioactivation, although the expression of CYP3A4 in the intestine of humans suggests that metabolism can also take place in the intestine [65,66,67]. The contribution of these enzymes to the metabolism of AFB1 in affected individuals depends on the expression level and affinity of the various enzymes; CYP3A4 may be most significant in *exo*-8,9-epoxide generation, and the relative contribution of CYP3A5, which also generates the *exo*-8,9-epoxide, differs individually [66]. The expression of CYP3A5 shows polymorphism and differs according to ethnicity. Such polymorphism could have an effect on the sensitivity to the toxic effects of aflatoxins [68]. CYP1A2 mostly results in the formation of hydroxylated metabolites of aflatoxin M1 and aflatoxin B1-*endo*-8,9-epoxide, which produce no DNA adduct. As aflatoxin crosses the placenta, interestingly, CYP3A7, a main CYP in the fetal liver of humans, can activate aflatoxin B1 to 8,9-epoxide [61]. Aflatoxin adducts were found in cord blood, showing that aflatoxin levels in the environment are biologically activated in utero to the reactive metabolites [69].

An important observation in the carcinogenicity of aflatoxins is the relationship between exposures and precise *TP53* tumor suppressor gene mutation in hepatocellular carcinoma (HCC) (liver cancer). In tumors from hepatocellular carcinoma patients in regions where aflatoxin is endemic, who were also affected by chronic hepatitis B virus (HBV), high incidence of a specific missense mutation, i.e., an Arg → Ser (AGG → AGT) point mutation at codon 249, has been reported in the gene [70,71]. This kind of mutation is very uncommon in hepatocellular carcinoma associated with hepatitis B virus in regions with a rare occurrence of aflatoxins, although it is not yet clear whether infection with HBV influences the occurrence of the HCC mutation in aflatoxin endemic regions. 

#### 3.1.2. Further Information about Ailments Caused by Aflatoxins

Detoxifying the aflatoxin *endo*-epoxide and *exo*-epoxide mainly occurs via conjugation mediated by glutathione *S*-transferase to reduced glutathione [3,72,73,74]. Furthermore, the *endo*-epoxide and *exo*-epoxide can be nonenzymatically hydrolyzed rapidly to aflatoxin B1-8,9-dihydrodiol, which then forms dialdehyde phenolate ions with an open ring. Dihydrodiol can react with the lysine’s ε-amino group in serum albumin, forming adducts of aflatoxin–albumin, which are commonly used as biomarkers to identify exposure [72]. In an additional metabolic stage, aflatoxin aldehyde reductase has been found to catalyze NADPH-dependent dialdehyde phenolate ion reduction to dialcohol (diol) [75]. The understanding of the induction of mutations, DNA damage, and metabolism in individuals with dietary exposure to aflatoxins contributes to the general evaluation of their adverse effects on human and animal health [72,73].

There were accounts of aflatoxin poisoning in human reported decades ago, although the earlier studies appeared inconclusive regarding the causative factors [67,76]. The affected patients presented jaundice after vomiting, anorexia, and fever, which worsened into edema and ascites in the lower extremities. There is evidence of aflatoxin poisoning resulting in patients presenting with low-grade fever, general malaise, anorexia, and abdominal discomfort, as well as tachycardia. In 2004, aflatoxicosis was reported in Kenya, a country in east Africa [34,77]. These outbreaks led to hundreds of deaths associated with the consumption of aflatoxin-contaminated maize. An aflatoxicosis case–control study, described as acute jaundice with no known origin, reported that levels of aflatoxins in foods from exposed households were far above the levels from foods obtained from unexposed households [77,78]. There were similar variations between controls and cases when blood levels of aflatoxin biomarkers were studied [77,78]. The association of aflatoxicosis and acute hepatitis with aflatoxin-contaminated maize has been sufficiently supported with evidence. Aflatoxicosis is mainly reported in regions that have maize as one of their staple foods. The aflatoxin intake levels associated with aflatoxicosis, as well as their causes of human death, have been studied [72]. Total aflatoxin levels causing fatality risks usually exceed 20 µg/kg (i.e., above 1 mg/day) bw per day in a normal adult. Other factors such as co-contamination with other toxins and an impaired immune system may influence the fatality rate. Aflatoxicosis with no fatality is also a possibility with 5–10-fold lower dosages. Additional estimations suggest that the AFB1 total intake linked to a 50% death rate in exposed individuals, i.e., the LD_50_ (median lethal dose), ranges from 0.54 to 1.62 mg/kg body weight, which is similar to the value of median lethal dose reported for baboons, pigs, dogs, cats, and rabbits [72]. The maximum residue level of AFM1 in milk is set to 50 ng/kg and 500 ng/kg of raw milk by the EU and the US, respectively. Consequently, the day-to-day exposure to staple foods, through the consumption of hundreds of grams a day and contaminated with at least 5000 µg/kg aflatoxins, could result in death in both humans and animals. Daily consumption of foods contaminated with at least 1000 µg/kg every day could result in aflatoxicosis. The levels of contamination found in maize, which are linked to aflatoxicosis or death, are 10–100 times the contamination levels regularly reported in several communities in sub-Saharan Africa. As maize being heavily contaminated with aflatoxins results in aflatoxicosis and even death, outbreaks of aflatoxins keep occurring. Therefore, there is a need to intensify efforts toward sensitizing people on how to avoid such outbreaks and limit the chances of exposure to aflatoxins, along with other mycotoxins.

The IARC classification of mycotoxins based on their carcinogenicity to humans is shown in Table 3. As the International Agency for Research on Cancer (IARC) classifies natural aflatoxin mixtures as being carcinogenic to humans (Group 1) [71], it is noteworthy to reiterate that aflatoxicosis can show combined effects with other infections such as HBV. A large case–control study reported that the combined effect of HBV infection and AFB1 exposure was more consistent with an additive model than a multiplicative one [79,80]. After an examination of plasma of cirrhosis patients, HCC patients, and controls for codon 249 AGG → AGT (the *TP53* gene mutation), it was shown that the higher risk linked to both HBV infection and 249 mutation was consistent with the multiplicative effect of chronic HBV infection and aflatoxin exposure [81]. The risk of HCC from aflatoxin exposure without chronic HBV infection has been difficult to assess in regions with widespread HBV infection. Omer et al. [82] stated that there is a 1.7–3.4-fold increased risk in those exposed to aflatoxins with no chronic HBV infection. Overall, the epidemiological evidence has shown a specific increased HCC risk from exposure to aflatoxins in people with chronic HBV infection, as well as considerable evidence of increased risk in people with aflatoxin exposure without chronic infection by HBV. Information is available on the relationship between aflatoxin exposure and liver cirrhosis. One case–control study carried out in the Gambia, a country in west Africa, concluded that increasing the lifetime intake of groundnut (considered one of the major grains affected by aflatoxin) was connected with a significantly increased risk of liver cirrhosis [83]. Although most countries allow low aflatoxin levels in peanuts and corns, some researchers have argued that these low levels can still result in damage to the liver in individuals affected by HBV who mostly depend on diets rich in grains, corn, and nuts. The presence of a codon 249 mutation, connected with aflatoxin, has also been associated with a comparable degree of increased risk of liver cirrhosis. Nevertheless, more studies, including controlled clinical studies, are required before drawing conclusions about liver cirrhosis and aflatoxins.

The immunomodulatory effects of aflatoxins have been studied experimentally in animals and cell models, as well as in observational studies involving farm animals [2,92,93]. Only few studies focused on the relationship between immune factors and aflatoxin exposure in humans, such as that conducted in Gambia [94]. Nonetheless, children highly exposed to aflatoxin would likely become susceptible to malaria parasitemia, although no significant correlations were revealed with malaria infection, lymphoproliferative responses, or antibody titer to *Plasmodium falciparum* asexual stages. Two studies conducted in Ghana compared the levels of aflatoxin biomarkers and peripheral blood cell subsets involving adults [95,96]. Higher levels of aflatoxin biomarkers were associated with lower levels of B lymphocyte antigens (CD19^+^ and CD3^+^) expressing the activation marker CD69^+^, as well as lower levels of CD8^+^ T cells expressing granzyme A and perforin [95]. The second study reported that higher levels of aflatoxin biomarkers were associated with lower levels of CD19^+^ cells expressing CD69^+^ and lower levels of CD8^+^ cells expressing perforin [96]. Additionally, HIV patients with high levels of aflatoxin biomarkers had significantly lower levels of CD4^+^ T regulatory cells and naïve CD4^+^ T cells in comparison to HIV patients with lower levels of aflatoxin biomarkers [96].

#### 3.1.3. Aflatoxin and Child Growth Impairment

Children face chronic exposure to high aflatoxin levels in regions with endemic food contamination. Exposure starts in utero, continuing throughout the early stages of life, although breastfeeding offers certain respite from high intakes per day. Studies carried out on many animals showed that exposure to aflatoxin has severe effects on development and growth [39]. Early studies examined the association between kwashiorkor and exposure to aflatoxin [76,97]. An early study linked the detection to aflatoxin in the blood of mothers with significantly lower birth weights of female babies [98]. Another study indicated a significant higher wasting prevalence in children that consumed cereals with high levels of aflatoxins, in comparison with children fed cereals with lower levels of aflatoxins [99]. Several studies have been conducted in children in west Africa with early exposure to aflatoxins. A cross-sectional study conducted on children within the age of 1 to 5 years in Togo and Benin reported a striking inverse relationship between growth and the level of aflatoxin–albumin adduct [39,100]. A longitudinal study conducted within 8 months reported strong negative correlations between an increase in height and the level of aflatoxin–albumin adduct [100].

The highest aflatoxin–albumin adduct quartile was linked to an average 1.7 cm decrease in height in comparison with the lowest quartile. In Gambia, a correlation was reported between aflatoxin exposure in utero and growth impairment in children within the first 12 months of life [69]. On the basis of these findings, the consumption of aflatoxin-contaminated foods by pregnant mothers may have significant effects on the growth and development of children after delivery. For children in regions grossly affected by aflatoxin contamination of food products, such as children in west and east Africa, growth impairment could occur during the introduction of solid foods, when there is a high likelihood of aflatoxin exposure. The dose–response associations between growth effects and levels of aflatoxin biomarkers are consistent with the causal effects. The action mechanisms through which aflatoxins might affect growth have not been fully established; however, compromised integrity of the intestine, via alteration in barrier function due to immune suppression or endothelial cell toxicity, is among the valid hypotheses that have to be properly studied [72]. In regions where aflatoxin contamination is prevalent, such as south Asia and sub-Saharan Africa, at least 7.1 million children below 5 years died in 2008 alone. Approximately 50% of these deaths (3.55 million) were associated with poor growth and undernutrition [101].

### 3.2. Ochratoxin A

Ochratoxins are mycotoxins with three forms of metabolites: ochratoxin A (OTA), ochratoxin B (OTB), and ochratoxin C (OTC). Ochratoxin A (OTA) is a toxic mycotoxin produced by *A. niger*, *A. ochraceus*, *Penicillium verrucosum*, and *A. carbonarius.* All ochratoxins are released by *Aspergillus* and *Penicillium* species. OTB is the non-chlorinated form of OTA, while OTC is the ethyl ester of OTA [40]. *A. ochraceus* is seen as a pollutant of several commodities including cereals, seeds, coffee, nuts, fruits, and dry meat, as well as beverages such as wine and beer. *A. carbonarius* is the major *Aspergillus* seen in vine fruit; it generates toxic substances during the process of juice production [41]. Ochratoxin A (OTA) is made by many *Aspergillus* and *Penicillium* species. OTA is among well-known food-borne mycotoxins. The infestation of agricultural commodities, including cereals and their products, dry vine fruits, spices, licorice, coffee beans, grape juice, roots, and wine, occurs globally. OTA is often formed during the crop storage in facilities and is generally known to cause some harmful effects in animals. Human exposure to OTA mostly occurs in regions that mainly eat foods produced from wheat and barley infested with causative fungi such as *P. verrucosum* and *A. ochraceus*. Minor sources include meat, particularly pork, obtained from animals that consumed infected grains. Bayman and Baker [40] and Mateo et al. [41] reported that OTA is a nephrotoxin and a carcinogen, directly linked to tumors in the human urinary tract, although there is limited research in humans due to confounding factors. The Joint FAO/WHO Expert Committee on Food Additives (JECFA) recommended a provisional tolerable weekly intake (PTWI) of ochratoxin A of 100 ng/kg body weight per week [2,102]. The risk assessment showed that OTA’s acute toxicity was reported in animals at lesser OTA levels than seen with long-term effects, including carcinogenicity; therefore, this provisional tolerable weekly intake is related to acute toxicity. Rare exposure to OTA, whereby contaminated foods are consumed once per week/month, can lead to persistent OTA blood levels [103]. Samples of blood from healthy individuals in Europe indicated OTA levels of 0.1 to 40 ng per mL [102]. While ochratoxin α is the main constituent identified in urine, the parent molecule remains the main compound detected in blood [103]. The most notable and sensitive effect of OTA is kidney damage (OTA is a nephrotoxin), but this mycotoxin can also have harmful effects on the immune system and fetus development [2]. Despite the clear evidence of kidney cancer and toxicity due to exposure to OTA in animals, the relationship in humans is not clear, although impacts on the kidney have been reported. In vivo, extensive differences in species exist in terms of the OTA serum half-life. OTA elimination in humans goes through two phases, fast excretion and slow clearance, with an estimated plasma half-life of 35 days.

#### 3.2.1. Mechanisms of Action of Ochratoxin A

Exposure to OTA is known to lead to acute toxicity in the kidneys of mammals. OTA exposure has been associated with the “Balkan endemic nephropathy”, a disease of the kidney with a high rate of mortality in those residing close to the Danube River tributaries in eastern Europe [104,105], although not all researchers agree on this. Allele carriers associated with phenylketonuria might have faced protection from miscarriage resulting from exposure to ochratoxin, suggesting a heterozygous benefit in spite of the likelihood of severe intellectual disability in uncommon cases of hereditary exposure from parents [104,105,106]. In mammals, ochratoxin A is absorbed in the GI tract and binds strongly to plasma proteins in the blood, mostly albumin, before being distributed to kidneys, with lower levels in the muscle, fat, and liver. Ochratoxin A is metabolized by various CYP enzymes, depending on tissue and species. In the human cells expressing CYP enzymes, 4(*R*)-hydroxy-OTA is the major metabolite and is formed by CYPs 2A6, 2D6, 2C9, 2B6, and 1A2, while of the 4(*S*)-hydroxy-OTA derivative is only formed by CYPs 2B6 and 2D6 [105]. Detected metabolites of OTA also include ochratoxin α and 10-hydroxy-OTA, formed by OTA peptide bond hydrolysis, resulting in no phenylalanine moiety and, thus, no toxicity. Moreover, OTA can be metabolized by lipoxygenase, epoxygenase, and cyclo-oxygenase, predominantly in extrahepatic organs including the kidney, yielding reactive oxygen species (ROS), which might cause oxidative damage.

Phenylalanine-tRNA ligase is competitively inhibited by ochratoxin A, causing protein synthesis inhibition and inhibition of the synthesis of DNA and RNA [105]. Phenylalanine coadministration can inhibit OTA acute toxicity in animals. DNA adduct formation by ochratoxin A and its possible role in the induction of cancer have been studied, with hypotheses suggesting the OTA-induced formation of direct adducts after metabolic activation through the phenoxy radical of OTA and indirect damage to DNA caused by the formation of oxygen radicals [105,106,107]. The concentrations of OTA-specific transporters in tissues were proposed to explain the relative sensitivities of species, target organs, and sex to the toxicity of ochratoxin A [108]. Another selective sensitivity contributor is the albumin binding degree that distinctly reduces OTA uptake by the transporter [109]. Mechanisms accounting for OTA carcinogenicity and toxicity without invoking OTA DNA adduct production have also been proposed, and they characteristically involve altering the expression of the genes that regulate cell death rates and cell proliferation rates [106]. Potential biomarkers of effects in the targeted tissues allow developing exclusive profiles of gene expression specific to altering OTA-induced gene expression. Genes include those involved in cell death, cell proliferation, cellular defense, and oxidative stress [104,110,111,112].

#### 3.2.2. Toxicities of Ochratoxin A

Evidence for ochratoxin A carcinogenicity mostly comes from experimental animal studies. Ochratoxin A is carcinogenic to mice and rats according to laboratory studies, leading to kidney carcinomas in rats and mice, and HCC in mice [40,41]. The action mechanism for OTA carcinogenicity is yet to be fully established. Descriptive studies have suggested an increased risk of cancer in people who consume ochratoxin A. The International Agency for Research on Cancer concluded that sufficient evidence exists to classify OTA as carcinogenic to laboratory animals but there is insufficient evidence to conclude that OTA increases the risk of cancer in humans. Therefore, the IARC classifies OTA as Group 2B, meaning that it is possibly carcinogenic to humans [2,89].

Short-term studies of OTA toxicity in pigs, dogs, rats, and mice evaluated the dose and timely development of progressive nephropathy. There were significant differences in species, sex, and administration route. Other toxicities of OTA include kidney lesions in chicken, myelotoxicities in mice, lesions of lymphoid tissues and the GI tract in hamsters, and hepatic and cardiac lesions in rats. Pigs are believed to be most sensitive to the nephrotoxic effects of OTA; the lowest observed level of effect (8 µg/kg body weight) was the basis used to establish the provisional tolerable weekly intake. Occupational studies conducted in European countries showed elevated levels of OTA in blood plasma samples in workers predisposed to grain dust exposure [105]. A grain farmer confined in a space contaminated with *A. ochraceus* suffered acute renal disease following temporary distress of the respiratory system [113].

### 3.3. Deoxynivalenol (DON)

DON is a trichothecene mycotoxin produced by fungal species such as *Fusarium graminearum* in cereals, such as wheat. *Fusarium* fungi are commonly seen in soil, where they release various toxins. Examples include trichothecenes such as nivalenol (NIV), T-2 and HT-2 toxins, deoxynivalenol (DON), fumonisins, and zearalenone (ZEA). *Fusarium* fungi and their toxins are seen in a range of cereals. Trichothecenes can have acute toxicity in humans, resulting in rapid irritation of the skin or intestinal mucosa, leading to diarrhea [2]. The chronic effects in animals include immune system suppression. The JECFA recommends 1 μg/kg bw/day PMTDI for DON on a no-observed-adverse-effect level (NOAEL) basis for a reduction in body weight with a 100-fold safety factor and 2 year bioassay in mice [114]. In mice, the NOAEL is 100 μg/kg body weight per day. DON exposure via inhalation is among the leading observation in many occupational evaluations relating to health hazards. In wheat, Fusarium head blight caused by *F. culmorum* or *F. graminearum* infection starts from the grain head exterior and passes into the interior. Accordingly, DON is mostly identified in the chaff and the kernel outer layers [115,116]. Grain dusts could have high DON concentrations and other mycotoxins in unsafe levels, which may not be the same in kernels.

#### 3.3.1. Mechanisms of Action of Deoxynivalenol

DON has direct toxicity through its epoxide moiety and consequently requires no metabolic activation for exerting its biological effect. Low-level exposure to trichothecene in animals modulates the expression of several chemokines and cytokines, which are key immune function regulators [116,117]. DON exposure results in upregulating mRNAs responsible for producing chemokines, cytokines, and other proteins associated with the immune system, which can also cause gene transcription. Additionally, deoxynivalenol modulates several mitogen-activated protein kinase (MAPK)-controlled physiological processes, including processes controlling cell apoptosis, cell differentiation, and cell growth, all of which are crucial for the immune response’s signal transduction [18,117]. Hence, along with the altered expression of cytokines, MAPK expression alterations can possibly contribute to dysregulation of the immune system and DON toxicity, as well as that of other trichothecenes. Activating processes resulting in a ribotoxic stress response are also connected to the DON activation of MAPK, and they are initiated by other inhibitors of translation, which, like deoxynivalenol, damage or bind to a specific region at the 28S rRNA 3′ end. Ribosomes play important roles in the ribotoxic stress response through functioning as a scaffold for the interaction between several MAPKs [18,116].

**Figure 2:**
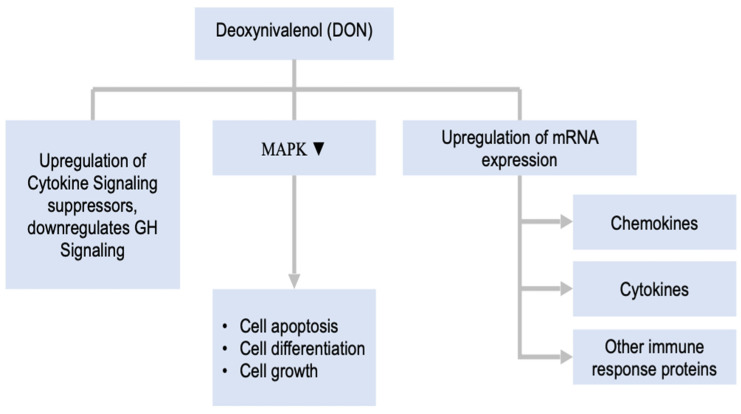
The mechanism of deoxynivalenol (DON) metabolism. MAPKs = mitogen-activated protein kinases. The downward-facing triangle beside MAPK indicates its alteration/decline retards the processes controlling cell apoptosis, cell differentiation, and cell growth.

The studies of DON toxicities revealed many approaches for the possible development of valuable biomarkers to identify its properties. For instance, exposure to DON in mice led to the upregulation of several cytokine signaling suppressors. These suppressors impair the signaling of growth s [118]. DON-induced growth hormone axis impairment precedes the retardation of growth in mice [119]. Oral administration of DON disturbs the growth hormone axis through the suppression of two growth-associated proteins, IGF1 and IGFALS. Therefore, the decreased expression of IGF1 and IGFALS, along with elevated levels of DON in urine, may serve as a potential biomarker for DON. This is illustrated in Figure 2, demonstrating how the mechanism of DON metabolism unfolds.

#### 3.3.2. Toxicities of Deoxynivalenol 

A few studies have reported DON carcinogenicity in experimental animals and humans. A bioassay was conducted in both sexes of B6C3F1 mice for 2 years, where they were fed deoxynivalenol in their diet at concentrations of 10, 5, 1, or 0 mg/kg; no increase in the incidence of neoplastic or pre-neoplastic lesions was reported in any tissue, including the liver [116]. DON is not known to be carcinogenic to humans. However, in humans, esophageal cancer was anecdotally associated with the consumption of grains contaminated with DON-producing *Fusarium* species [89]. The IARC resolved that there is insufficient evidence in experimental animals and humans to determine DON carcinogenicity. Therefore, the IARC categorizes DON as Group 3, meaning that it is not classifiable as carcinogenic to humans [89]. DON has demonstrated many toxicities in animals, such as immunotoxicity, teratogenicity, cardiotoxicity, gastroenteritis, reduced weight gain, and feed refusal [119,120,121]. DON has been shown to cause acute toxicities in humans, with the main symptom being severe GI toxicity. Consumption of DON-contaminated cereals was linked to several incidents of poisoning in China and at least one outbreak in India [120]. In the outbreaks, symptoms observed included fever, dizziness, headache, diarrhea, abdominal pain, vomiting, nausea, and rapid onset. DON poisoning in China was associated with DON-contaminated wheat (0.3 to 100 mg/kg DON levels) [120].

### 3.4. Fumonisins

Fumonisins are mycotoxins produced by *Fusarium* species, including the section Liseola. Structurally, they are strongly similar to sphinganine, the sphingolipid backbone precursor. Fumonisins B1, B2, B3, and B4 (FB1, FB2, FB3, and FB4, respectively) are the most common fumonisins. Over 15 fumonisins have been described to date with other minor metabolic compounds reported, although it has not been shown whether many occur naturally [43]. In 2015, an exceptional class of non-aminated fumonisins was reported on *Aspergillus welwitschiae*-infected grapes, although their toxicology is yet to be fully established [46]. Fumonisins are mostly found in maize and less frequently in other cereals. The JECFA established a provisional maximum tolerable daily intake (PMTDI) of 2 μg/kg body weight per day for fumonisins B1, B2, and B3 alone or combined [2,92,102]. This JECFA PMTDI is based on the NOAEL for nephrotoxicity of 0.2 mg/kg body weight per day in rodents, divided by a 100-fold safety factor; it is not based on tumorigenicity data [92].

#### 3.4.1. Mechanisms of Action of Fumonisins

**Figure 3:**
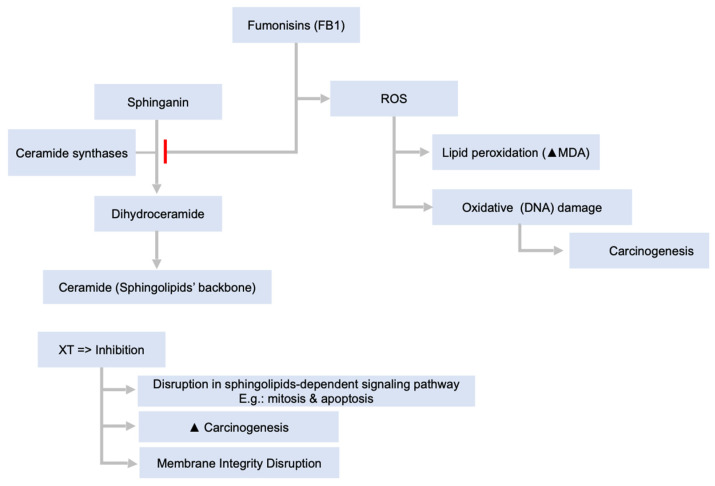
The action mechanisms involving the breakdown of fumonisins. The red line indicates that both Sphinganin and Ceramide synthases are needed for dihydroceraminde. The upward-facing arrow beside MDA (malndialdehyde), shows it increases lipid per oxidation; The upward-facing arrow beside carcinogenesis shows the chances of its occurrence increases with ceramide presence.

Figure 3 shows the action mechanisms involving the breakdown of fumonisins. In particular, fumonisins exhibit their actions via various mechanisms, most of which are fully established, whereas others are either still under establishment or yet to be explored. The genotoxicity of FB1 is not fully understood, although in vitro studies showed that fumonisin B1 induced chromosomal aberrations and micronuclei [71,122,123]. The damage to the DNA may be due to the stimulation of lipid peroxidation and oxidative damage, which is consistent with the increase in malondialdehyde adducts and DNA oxidative damage of kidney and liver in rats and with lipid peroxidation in vivo after treatment of FB1 [124,125]. Liver carcinogenesis induced by fumonisin proceeds via the stages of initiation and promotion similarly to genotoxins, and it is time- and dose-dependent [126]. Fumonisin B1 disrupts the de novo synthesis of sphingolipids by inhibiting ceramide synthase, leading to several effects on cell functions and signaling pathways which depend on ceramide, complex sphingolipids, sphingoid base 1-phosphates, and sphingoid bases [127], including effects on mitosis and apoptosis, thereby possibly aiding carcinogenesis via altering the balance of cell replication and death [123]. The disruption of sphingolipid metabolism results in changes in the ratio of sphinganine to sphingosine, with an increase in the concentration of sphinganine in tissue, closely correlating with the in vivo carcinogenicity and toxicity of fumonisins [128]. Disrupted synthesis of fatty acids, phospholipids, and cholesterols, and their interaction with ceramide are thought to play an important role in the altered differential growth patterns of hepatocytes during the promotion of liver cancer [129]. In experimental animals, the role of hepatocytes in immunomodulation was thought to be via changes in the levels of cytokine, in vivo and in vitro, as well as via effects on responses to antibody vaccines in pigs infected with fumonisin B1 [123,125,128,130].

#### 3.4.2. Toxicities of Fumonisins

Studies have shown that contamination with both fumonisins and *F. verticillioides* in maize positively correlates with cancer of the esophagus in rats [35,45,71,131]. A study reported that the urinary ratio of sphinganine to sphingosine increased significantly in males that consumed more than 110 µg FB1/kg body weight per day [132,133]. Nevertheless, subsequent studies reported no relationship between ratios of sphinganine to sphingosine or sphingoid bases in urine and plasma and individual exposure to fumonisins, showing that these biomarkers may be insufficient and insensitive for monitoring human exposure to fumonisins [134,135,136]. There are synergistic interactions between FB1 and AFB1 in liver cancer development [133,137,138]. Considering these interactions, the co-contamination of fumonisins and aflatoxins in foods, and the occurrence of both myctoxins in populations with high HBV infection prevalence, fumonisins could play a plausible role in HCC [45,139]. The IARC placed FB1 and FB2 in Group 2B, classified as possibly carcinogenic to humans [71,89].

#### 3.4.3. Neural Tube Defects and Fumonisins

Studies conducted on animals showed that exposure to fumonisins can result in neural tube defects, most likely via disrupting the biosynthesis of sphingolipids and subsequent sphingolipid depletion, which are important for the function of lipid rafts, particularly folate processing through folate transporters with high affinity [44,140]. Neural tube defects usually feature reduced levels of folate, and disruption of the cell membrane caused by fumonisins may result in reduced absorption of folate via impairment of the membrane folate receptors [141]. The elevation of sphingoid base 1-phosphates caused by exposure to fumonisins is associated with neural tube defects [142].

### 3.5. Zearalenone (ZEA)

Zearalenone, also known as F-2 mycotoxin, is a nonsteroidal estrogenic metabolite produced by some *Fusarium* and *Gibberella* species, such as *Fusarium graminearum* [3,47]. ZEA may occur along with DON, especially in maize, rice, millet, sorghum, rye, oats, barley, wheat, etc. Zearalenone is distributed globally [143]. Dietary sources of human exposure per day are estimated to be between 1 ng/kg and 30 ng/kg body weight [143,144]. The JECFA established a PMTDI of 0.5 µg/kg body weight per day for ZEA; this estimate was due to the NOAEL for hormonal effects in pigs [145]. ZEA was demonstrated to have hormonal and estrogenic effects, resulting in infertility following high amounts of ingestion, especially in pigs. Fumonisins are associated with esophageal cancer in humans and with kidney and liver toxicity in animals.

#### 3.5.1. Mechanisms of Action of Zearalenone

**Figure 4:**
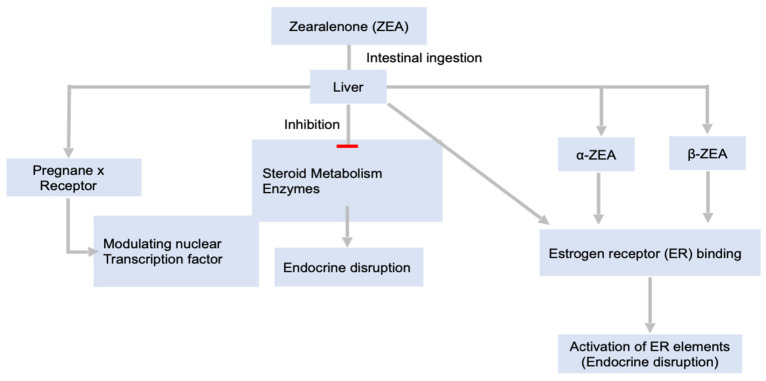
The action mechanisms involving zearalenone (ZEA). α-ZEA = α-zearalenol; β-ZEA = β-zearalenol. The red line signals liver functions to inhibit ZEA in competing with the steroid metabolism enzymes.

Figure 4 shows the action mechanisms involving zearalenone (ZEA). In particular, ZEA is metabolized in pigs during intestinal tissue absorption. Importantly, the metabolism of ZEA involves the reduction of its 6-keto group, leading to α-zearalenol and β-zearalenol formation and, upon additional reduction, α-zearalanol and β-zearalanol, all of which can be conjugated to glucuronic acid [145,146]. In vitro studies on liver microsomes suggested that the high rate of α-zearalenol production in comparison to β-zearalenol in humans and pigs is important due to the higher relative estrogenicity of the former in comparison to zearalenone [147]. Thus, α-zearalenol formation may be a contributing step to bioactivation of the ZEA estrogenic effects. Zearalenone and its metabolic derivatives may bind to receptors of estrogen, leading to several changes to binding nucleus elements responsive to estrogens. Additionally, zearalenone is a competing substrate for enzymes involved in the metabolism and synthesis of steroids; thus, it could be an endocrine disruptor. Zearalenone can bioactivate the pregnane X receptor through recruiting coactivators and displacing corepressors [148]. Consequently, zearalenone may have wide effects on the expression of genes by modifying the activities of nuclear transcription factors. 

#### 3.5.2. Toxicities of Zearalenone

Zearalenone can cause an increase in the incidence of pituitary tumors and liver cells in mice, in line with the hormonal mode of its carcinogenic actions [89,149]. Elevated serum levels of α-zearalenol and ZEA are associated with early puberty [42]. The IARC placed ZEA in Group 3, i.e., not classifiable as carcinogenic to humans. A high incidence of esophageal cancer is found in regions with a high contamination of mycotoxins, including zearalenone, and ZEA’s ability to induce hyperkeratotic papilloma in the rat esophageal squamous epithelium forestomach suggests its involvement in tumor development in the gastrointestinal tract [150]. In the treatment of cultured mouse bone marrow and Vero cells with ZEA, ZEA induced micronuclei formation, as well as induced genotoxicity and clastogenicity [151]. ZEA has the ability to attack DNA [42].

In C3hA^vy^fB mice, ZEA induced mammary tumors [152], but further studies are required to determine whether ZEA has the ability to induce cancer or has carcinogenic effects on some specific organs such as steroidal estrogens and diethylstilbestrol [153]. In female mice, zearalenone was reported to induce hepatocellular adenomas [42]. ZEA was reported to cause damage to *Bacillus subtilis* DNA, showing DNA adducts in the liver and kidney of female mice [154]. ZEA has been shown to be genotoxic, with the ability to cause hepatocellular adenomas in mice [123,153]. Zearalenone was reported to be both genotoxic and carcinogenic by affecting hormonal activities in several animals [155]. Zearalenone could induce modifications in the reproductive systems and organs of several experimental animals, including rats, mice, and various domesticated animals [42,156]. Zearalenone was found to be carcinogenic in mice and capable of causing hepatocellular adenomas and pituitary tumors [153].

### 3.6. Other Essential Mycotoxins 

#### 3.6.1. Patulin

Patulin is released by *P. expansum*, *Penicillium*, *Paecilomyces*, and *Aspergillus* species. *Penicillium expansum* is mostly reported to occur in many moldy fruits and vegetables and grains, especially rotting maize, peanuts, apple, fig, acha, etc. [38,155,157]. Fermentation is known to destroy patulin; consequently, it is not in fermented apple beverages, e.g., cider. Patulin has not been found to be a cancer-causing agent; however, it was found to harm the immune system of animals [155]. The European Community in 2004 set limits to patulin concentrations in food products; currently, these are 10 μg/kg for apple-related products (apple juice inclusive) for infants and children, 50 μg/kg in fruit juice, and 25 μg/kg in products of solid apples intended for straight intake for adults [155,157]. Although patulin is commonly detected in decaying apple and its products, it also occurs in many fruits, grains (mostly cereals and legumes), and other common foods. The major dietary sources of patulin in human are apple and its juices made from contaminated fruits [2]. The acute symptoms of patulin include liver and kidney toxicity, spleen damage and toxicity, and immune toxicity. In humans, gastrointestinal (GI) disturbances, vomiting, and nausea are usually reported. Patulin is genotoxic, but the potential for carcinogenicity is yet to be reported. 

Patulin has a precursor, known as 6-methylsalicylic acid; together, they are derivatives of acetyl-CoA, making them polyketides and possible carcinogens [42]. Patulin and 6-methylsalicylic acid can cause gene mutations in various cells of mammals [158]. A 15 month subcutaneous patulin administration two times per week indicated malignant tumor cell development in the administration area, showing the carcinogenic effect of patulin [42]. Patulin has also shown toxicity in mice born to mothers given patulin; deaths were reported in both females and males. Patulin is teratogenic, carcinogenic, and mutagenic, and it can cause injuries to the intestine, as well as impair cellular DNA in humans and bacteria, leading to cancer and tumor development [42,159]. It has been suggested that patulin toxicity in intestinal cells occurs by inactivating the active site of protein tyrosine phosphatase (PTP). PTP is an important regulator of the function of the intestinal epithelial barrier. The PTP active site has a cysteine residue (Cys215) required for phosphatase activities. However, sulfhydryl reacting compounds, e.g., acetaldehyde, reduce transepithelial resistance (TER) via covalently modifying the PTP’s Cys215, which may result in intestinal cell damage and eventually cause stomach and intestinal cancers [18,35,159].

#### 3.6.2. Citrinin

Citrinin is a mycotoxin first seen in the mold *Penicillium citrinum*. It has been reported in more than 12 *Penicillium* species and numerous *Aspergillus* species. In 2003, Bennett and Klich reported that citrinin is associated with the yellowed rice disease reported in Japan, and it also acted as a nephrotoxin in all tested animal species. Although citrinin is connected to several agricultural crops, such as barley, oats, rye, rice, corn, and wheat, as well foods colored using the *Monascus* pigment, its total implication for humans is not yet known. Citrinin is reported to work synergistically with OTA in impairing RNA synthesis in murine kidney [25].

#### 3.6.3. Ergot Alkaloids

Ergot alkaloids are chemical substances released as toxic mixtures of alkaloids in the sclerotia of *Claviceps* species that are known pathogenic microbes of many species of grasses. Ergot sclerotia ingestion from infected cereals, commonly in the form of bread made from contaminated flour, results in ergotism, a human disease known as St. Anthony’s Fire [25]. The two forms of ergotism are convulsive, which has effects on the central nervous system (CNS), and gangrenous, which is known to affect blood supply to the extremities. Ergot alkaloids induce ergotism and low nerve fever, with strong effects on fertility in humans [160,161]. Bennett and Klich reported that modern grain cleaning methods have significantly reduced ergotism as a human disease; however, it is still an important veterinary problem [25].

#### 3.6.4. Trichothecene Mycotoxins

##### T-2 Mycotoxin

T-2 is a trichothecene mycotoxin that has lymphocytic, carcinogenic, cytotoxic, and immunosuppressive actions against mammalian cells [50,162]. It can induce breaks in lymphocyte DNA *in vitro* and *in vivo*. When fibroblast cells are treated with T-2 combined with ^3^H-thymidine, there is unscheduled synthesis of DNA [49,162]. T-2 toxin has cellular effects on the culture models of primary hepatic cells of chickens [163]. T-2 toxin induced apoptosis and developmental toxicity in zebrafish embryos [48]. The susceptibility to T-2 of immature animals and newborns can be compared to that of adults; it can cause dermal toxicities and edema via direct attack on capillary vessels. T-2 induced liver injuries in New Zealand rabbits, and it has also been shown to induce intestinal injury [164,165]. In vitro, T-2 can reduce the response to mitogens in human lymphocytes [156].

##### Diacetoxyscirpenol (DAS)

Diacetoxyscirpenol (DAS) or 4,15-diacetoxyscirpenol (DAS), also known as anguidine, is a trichothecene mycotoxin secondary metabolite produced by the *Fusarium* genus, which has been shown to be toxic to animals and humans. DAS inhibits the production of Ig in human lymphocytes stimulated by mitogen and can cause esophageal hyperplasia [52,53]. There is insufficient information on the toxicokinetics and toxicity of DAS in farm and experimental animals. As a result, human chronic and acute health-based guidance values (HBGV) are estimated using data generated from DAS clinical trials as an anticancer agent following intravenous administration in cancer patients, as well as using hazard characterization after exposure via the oral route. The major adverse effects following repeated and acute exposure were hematotoxicity, with the NOAEL for DAS being 65 μg/kg body weight, and emesis, with the NOAEL of DAS being 32 μg per kg body weight [51]. A 0.65 μg/kg bw tolerable daily intake (TDI) and a 3.2 μg/kg bw acute reference dose (ARfD) have been established for DAS [51]. The highest average chronic and acute dietary exposures in the Europe were noted to be 0.49 and 0.8 μg/kg bw/day, respectively; these values pose no health concerns to humans. There is limited information regarding the effects of DAS on pigs, dogs, poultry, chicken, etc. [51]. More studies are required to firmly establish whether DAS has more toxicities beyond what is currently known.

##### Fusarenon X (FusX)

Fusarenon X (FusX) is a trichothecene capable of causing cytotoxicity, carcinogenicity, and immunosuppressive response in animal models and possibly in humans. It has been shown to be toxic to several types of cells, including murine thymocytes, gastric epithelial cells, and lymphocytes, along with a high toxicity to human hepatoblastoma cells [54,56,166]. In vitro and in vivo, FusX initiates apoptosis in mouse thymocytes, which may be hypothetically applicable to humans [53,55]. Fusarenon X has been shown to be carcinogenic, especially to animals [54]. It is very cytotoxic to many cells; it is also believed to have chromosomal effects and to be teratogenic. FusX can be genotoxic even at low levels [55]. Additionally, with regard to genome science, FusX is a quick one-step transcription activator-like effector assembly system [167]. The FusX TALE Base Editor has been studied and reported for quick mitochondrial DNA programming of zebrafish disease and human cell models in vivo and in vitro, respectively, [56].

##### Nivalenol (NIV)

Nivalenol (NIV) is a trichothecene mycotoxin, which, in nature, is mostly produced by species of *Fusarium*. Nivalenol, T-2 toxin, and DON were used as bioweapons in some places such as Laos, Cambodia, and Afghanistan, and they were all detected in the vegetation at sites affected, while the T-2 toxin was also detected in the blood and urine samples of victims [58]. NIV can increase the rate of induced cancer and mutation. It also played a role in the exchange of sister chromatids in the cells of Chinese hamsters and could cause DNA damage, which makes nivalenol potentially genotoxic [52,53]. 

NIV causes changes in several biological pathways, of which the NF-κB (nuclear factor kappa-light-chain-enhancer of activated B cells) pathway is the most common and possibly significant. NF-κB, a transcription factor, is seen in nearly every cell in humans, and it regulates the expression of its target genes through binding to specific genomic DNA (gDNA) motifs on regulatory elements [57]. NIV can change cytokine expression and induce IL-8 secretion; MCP-1/CCL2 is another factor affected by NIV [57]. NIV can cause reduced CCL2 secretion and, consequently, reduced monocyte mobility. This shows that NIV is immunosuppressive. NF-κB and NIV interact to influence cells [168]. While DON induces the secretion of immunorelevant messenger molecules called chemokines, NIV inhibits their secretion [169]. Furthermore, NIV upregulates the expression of proinflammatory genes in macrophages, showing mixed effects on various types of cells, even at a cytotoxic level. Another NIV cytotoxic mechanism is cytotoxic apoptosis, which indicates that NIV could be more toxic than DON, its usually co-occurring mycotoxin, via inducing DNA damage/apoptosis [170]. NIV also changes the proliferation rate of human leukocytes in a dose-dependent manner. Lower concentrations improve the proliferation of leukocytes, while higher concentrations reduce their proliferation in a dose-dependent manner [171]. Nivalenol affects the genes of Chinese hamster V79 (CHO) cells through a slight increase in the frequencies of sister chromatid exchange and chromosomal aberrations [57]. It led to DNA damage in mice and in Chinese hamster V79 cells. In mice fed intraperitoneally with 3.7 mg nivalenol/kg body weight or orally with 20 mg/kg body weight, there was DNA damage in the colon, jejunum, stomach, bone marrow, and kidney [57]. The DNA in the liver and thymus was unaffected. In DNA-damaged organs, upon histopathological examination, no necrotic changes were observed. Acute toxicity of NIV induces toxicities of lymphoid organs and bone marrow. Prolonged exposure can cause leukopenia and erythropenia. It was observed in mice that NIV increased serum IgA presence, accompanied by changes in immunopathology in kidneys analogous to IgA nephropathy in humans; in mice and rats, NIV exhibited toxicity with leukopenia and growth retardation with dosages as little as 0.7 mg/kg body weight per day [57]. Lethal doses depend on the administration/intake route. As NIV is often given with feed, the oral administration LD_50_ (19.5 mg/kg body weight per day and 38.9 mg/kg bw per day in rats and in mice, respectively) could serve as the standard. The subcutaneous (SC), intraperitoneal, and intravenous LD_50_ ranges from 7 to 7.5 mg/kg body weight per day [57]. Nivalenol chronic toxicity can result in leukopenia [55,163]. In the presence of AFB1, nivalenol enhances AFB1-induced hepatocarcinogenesis.

## 4. Some Mycotoxins Are Directly Involved in Some Types of Cancer 

### 4.1. Breast Cancer

There has been conflicting information about the association between breast cancer and ZEA [172,173]. A study done in north Africa reported a likely role of α-zearalanol (α-ZAL) in the development of breast cancer [173]. α-Zearalanol can originate from the metabolism of ZEA or from the consumption of foods; however, it is not yet an entirely characterized vector as α-zearalanol is also seen in meat when used in cattle for promoting growth [174]. Its diastereomers zearalanol (ZAN) and β-zearalanol (β-ZAL) are α-ZAL metabolites formed after human ingestion. Additionally, α-zearalanol can be conjugated with sulfonic acid or glucuronic acid [173]. ZEA is structurally similar to estradiol, a hormone; thus, it exerts affinity toward estrogen receptors and may affect fertility in livestock and humans. Various in vivo estrogenic potential effects have been reported for zearalenol and its metabolic compounds. In order to take this these differences into account, ZEA molar potency factors (relative potency factors (RPFs)) were estimated and applied to estimates for exposure to various ZEA metabolites. RPFs were molecularly administered for ZEA and its metabolic compounds (reference 1.0) according to the EFSA CONTAM Panel recommendation, with α-zearalenol (α-ZEL) RPF 60 and α-zearalanol RPF 4.0 [175]. All these discoveries suggest that ZEA and its metabolic compounds could play key roles in the cancer of reproductive organs in animals and humans [42,172]. ZEA is carcinogenic in mice, where it causes pituitary tumors and hepatocellular adenomas [154]. More epidemiological studies and clinical assessments are required to establish whether mycotoxins such as zearalenol have a potential role in breast cancer in humans and animals. 

### 4.2. Liver Cancer (Hepatic Cancer)

Aflatoxins such as AFB1, AFB2, AFG1, AFG2, and AFM1 are characterized as Group 1 carcinogens [85,86]. According to the IARC report, aflatoxins are annotated as medium-priority agents with possible consideration for future evaluation regarding additional sites of cancer [176]. Several studies have confirmed the link between aflatoxins and increased risk of hepatic cancer. Consumption of aflatoxin-contaminated foods has also been linked with a high risk of liver cancer [177]. Studies conducted in animals reported the carcinogenic effects of AFB1 and AFG1, in contrast with AFG2 and AFB2, where insufficient evidence on carcinogenicity exists. The organ most targeted by aflatoxins is the liver, with liver damage reported in fish, nonhuman primates, rodents, and poultry given AFB1. During mitosis, AFB1 induces genetic recombination, point mutations, and genetic instability in mammalian cells at molecular levels. AFB1 causes mutagenicity via a direct action of genotoxicity [85,178]. Aflatoxin B1 is converted to aflatoxin-8,9-epoxide (highly reactive and unstable form) through cytochrome P450 (CYP450) oxidation, which can bind to proteins (e.g., albumin) or DNA [42,179,180]. Aflatoxin-8,9-epoxide reactions with DNA molecules results in the formation of the aflatoxin-N7-guanine-adduct, which leads to G:C to T:A transversion mutations during DNA replication [181,182]. If the mutations take place in tumor suppressor genes or oncogenes (vital cancer-related genes), they may result in increased abnormal cell proliferation, leading to cancer development.

Aflatoxins are linked to the incidence of HCC in low-income and middle-income nations, through consuming agricultural crops mostly produced using subsistence farming [183,184]. The association between aflatoxins and liver cancer has been mostly studied with respect to HCC, suggesting that various causes of different liver cancer subtypes, e.g., unspecific PLC, cholangiocarcinoma, and HCC, may explain the heterogeneous results from different studies [177]. Factors that influence the risk of liver cancer include obesity (or overweight), consumption of alcohol, liver cirrhosis, chronic use of oral contraceptives with high estroprogestative agents, consumption of foods contaminated with aflatoxins, chronic hepatitis B/C, and smoking. Synergistic effects of aflatoxin exposure and HBV infection may be described by a virus-induced increase in CYP450, converting aflatoxin to its reactive metabolic compound [177].

Statistically, no significant association was reported between FB1 exposure and HCC. FB1 can alter protein synthesis and, in vitro, can inhibit DNA synthesis in intestinal cells in high concentrations [185,186]. A bioassay in rats reported FB1 hepatotoxicity and hepato-carcinogenicity [187]. There is a recommendation for a high evaluation priority for FB1 due to substantial emerging information after the previous evaluation by the IARC Monographs [176]. There is evidence for ceramide synthase inhibition in individuals who consumed corn-based foods high in FB1 [188]. Urinary fumonisin B1 can be used for assessing current FB1 exposure in population-based studies. Additionally, the elevation of phosphorylated sphingoid bases in embryonic fibroblasts of FB1-treated mice is associated with increased histone lysine acetylation and reduced histone deacetylase activities [88,189]. Ceramide synthase inhibition by FB exposure leads to increased serum levels of sphingolipids, which can serve as a biomarker for mycotoxin exposure [127,190]. As a result of a lack of validated biomarkers for FB1 exposure, based on rodents, a 128 min serum half-life in humans was allometrically projected; few studies in humans have studied HCC’s association with FB1 [190,191,192]. 

### 4.3. Cervical Cancer

It has been hypothesized that zearalenol can induce genital cancer in humans, as it has estrogenic activities in several animal models, and it also forms DNA adducts in the genitalia of rats, mice, and other animals, including horses [172,184]. Zearalenone may have wide effects on gene expression by modifying the activities of nuclear transcription factors. ZEA can attack the DNA. More quality studies are required to firmly establish the relationship between cervical cancer and ZEA, as well as other mycotoxins.

## 5. Actions of Mycotoxins on Human Cellular Genome: A Primer

AFB1 and OTA are the most toxic mycotoxins to animals and humans. AF1 is the most carcinogenic mycotoxin and can penetrate cell membranes, subsequently attaching to DNA where it causes genomic modifications. AFB1 is liposoluble and is absorbed from exposure sites in the blood stream [193,194,195,196]. As outlined in previous sections, aflatoxins are metabolized by CYP450 enzymes, once they enter cells, to the unstable and highly reactive aflatoxin-8,9-epoxide, which then binds to DNA or protein molecules for more stability [197,198]. As soon as aflatoxin-8,9-epoxide is highly bound to DNA, it results in the formation of aflatoxin-N7-guanine, which causes GC–TA transversion mutations. The cell cycle is directly affected via effects on the P53 gene that encodes the tumor suppressor protein, which inhibits cancer and tumor development [199].

Citrinin is among the strongest nephrotoxins to animals, and its toxicity levels vary according to species. In rodent kidneys, citrinin acts synergistically with OTA to interfere with and suppress RNA synthesis. Citrinin inhibits the expression of cytokines and reduces interleukin-4 in T-helper type 1, possibly leading to increased risk of allergies in human [200]. At high concentrations, citrinin is genotoxic toward cultured human lymphocytes [201]. Citrinin is a nephrotoxin that imposes oxidative stress and causes nephropathy, as well as increases the mitochondrial membrane permeability [202]. 

Ochratoxin targets the kidney. OTA is nephrotoxic to humans and animals, and it is considered a potent hepatotoxin, carcinogen, immune suppressant, and teratogen [203]. OTA interrupts cellular physiology in several ways, but its primary effects are often linked to enzymes involved in phenylalanine metabolism by inhibiting enzymes involved in phenylalanine-tRNA complex synthesis. OTA can inhibit the production of mitochondrial ATP, as well as stimulate lipid peroxidation.

FB1 is carcinogenic and hepatotoxic, while it can also cause liver apoptosis and possibly esophageal cancer [204]. It disrupts sphingolipid metabolism by inducing lipid peroxidation which alters the cell membrane, resulting in cell deaths via apoptosis. FB1 can also inhibit DNA synthesis and protein synthesis, especially at high concentrations [186]. It exhibits its carcinogenic activities via ^3^H-thymidine incorporation. Fumonisin exposure in animals contributes to neural tube defects, and it has been hypothetically suggested to have similar effects in humans. Several cases of spina bifida and anencephaly in Texas, USA, were associated with fumonisin-contaminated corn-based foods [205]. The IARC classifies fumonisins as Group 2B, i.e., possibly carcinogenic to humans (refer to Table 3). In general, mycotoxin removal from foods and feeds has some challenges, which means that their presence is a serious health concern [206]. Trichothecenes have been shown to have multiple inhibitory activities on eukaryotes by inhibiting DNA synthesis, mitochondrial function, RNA synthesis, and protein synthesis, as well as by affecting cell membrane and cell division [207,208]. Trichothecene mycotoxins induced programmed cell death responses in affected cells, and they could decrease downstream gene products, activate protein kinase with mitogens, and initiate the ribotoxic stress response [209].

## 6. Concluding Remarks and Future Prospects

Understanding the mycotoxins’ toxicological mechanisms associated with humans and animals still remains an important aspect of public health and environmental concern. Through this synthesis, we have learned more about the mycotoxins’ toxicological mechanisms associated with humans and livestock. In particular, we have deepened our understanding about how the mycotoxins’ toxicological mechanisms impact on the human cellular genome. For emphasis, the major mycotoxins associated with foods, humans, and livestock, including aflatoxins, citrinin, ochratoxins, fumonisins, patulin, zearalenone, nivalenol, deoxynivalenol, fumonisins, and ergot alkaloids, must be taken very seriously. We also demonstrated that a number of these mycotoxins can be carcinogens, nephrotoxins, mutagens, etc., as they can retard human growth and wellbeing. While both aflatoxin B1 and ochratoxin A remain among the very toxic mycotoxins associated with animals and humans, it should also be noted that majority of mycotoxins generally exhibit potent toxicities to different degrees. Nonetheless, the risks of liver cancer, breast cancer, cervical cancer, esophageal cancer, etc. remain a very serious global concern, as they have been linked to exposure to mycotoxins such as aflatoxins, ochratoxins, zearalenone, fumonisins, citrinin, and deoxynivalenol. DNA damage, kidney damage, DNA and RNA mutations, growth impairment in children, gene modifications, and immune impairment, as well as the inhibition of DNA synthesis, mitochondrial function, RNA synthesis, protein synthesis, and cell division, have also been linked to exposure to mycotoxins.

An area that has not been fully captured in this contribution is the quantitative information about the transition of mycotoxins from animal feeds to meat, milk, eggs, or other edible parts of livestock and poultry, and this should be the direction of a future review study. Considering that animal products are potentially contaminated by mycotoxins in feed, the toxicity of human foods is apparently dependent on their concentration. Another area that requires additional review synthesis is knowledge underscoring how some mycotoxins particularly fusarium toxins are able to cause food/feed intake reductions and to understand this phenomenon is needful, especially the potential effects (of mycotoxins food/feed intake). Further studies are required to establish the mechanisms underlying the environmental implications of mycotoxins in the course of affecting humans and animals. More risk assessment studies are also required, especially in evaluating the factors and conditions that facilitate the toxicities of mycotoxins.

## Figures and Tables

**Table 1:** Summary of recently conducted reviews on mycotoxin toxicology involving humans and livestock, as well as control/removal strategies.

Objectives of Literature Review	Key Sections	References
This review discussed mycotoxin toxicities from the perspective of consumer health safety concerns, as well as mitigation/treatment strategies	Toxicology, consumer health safety concerns, and actions of mycotoxins; toxic effects of combined mycotoxins exposure; major mycotoxin effects on infants and children; complications/risks of mycotoxin exposure at various stages of human life; consumer health implications of mycotoxin exposure; mitigation/removal strategies of mycotoxin toxicities	Awuchi, Nwozo, et al. [5]
This review introduced the natural occurrence of *Alternaria* mycotoxins, as well as their toxicity, metabolism, and analytical methods	Toxicity of *Alternaria* mycotoxins, metabolism of *Alternaria* mycotoxins, *Alternaria* mycotoxin analysis	Chen, Mao, et al. [10]
This review revisited how mycotoxins affect animals, foods, humans, and plants, specific to types, toxicity, prevention measures, and strategies for detoxification and removal	Major groups of mycotoxins: occurrence, production, and toxicities; mycotoxin prevention, decontamination, and detoxification approaches	Awuchi, Ondari, et al. [18]
This review discussed the co-occurrence of masked mycotoxins, as well as their sampling and extraction, and the suitability of LC–MS/MS for accurate and precise analysis/detection	Recent challenges in the analysis of mycotoxins; analytical techniques and extraction of mycotoxins from food samples	Iqbal [11]
This review summarized the occurrence of mycotoxins, their toxic effects, and the detoxifying agents with emphasis on deoxynivalenol in pig production	Mycotoxin occurrence; mycotoxin toxicity; mycotoxin-detoxifying agents	Holanda and Kim [21]
This study reviewed the information reported on the toxic effects of the most relevant/studied *Fusarium* toxins and their modified forms over the last few year	Metabolism of DON, T-2, HT-2, and ZEN toxins, as well as their modified forms	Pierzgalski et al. [14]
This review comprehensively summarized the latest (target and nontarget) knowledge of qualitative and quantitative analysis for modified mycotoxins, elucidating their major transformation mechanisms	Status of global mycotoxin contamination; transformation of the modified mycotoxins; analysis strategy of modified mycotoxins and metabolites; challenges in modified mycotoxins	Lu, Qin, et al. [15]
This review summarized the occurrence and toxicological aspects of major *Aspergillus*-derived mycotoxins	Food toxicology and molecular mechanism of mycotoxins; occurrence of *Aspergillus*-derived mycotoxins in the feed and food chain; prevention strategies of mycotoxicoses; medical aspects of *Aspergillus*-derived mycotoxins	Ráduly, Szabó, et al. [17]
This review provided the most important mycotoxins in crops/finished fish feed, i.e., aflatoxins, fumonisins, ochratoxins, trichothecenes, and zearalenone	Mycotoxin contamination of fish feed; aflatoxins and their precursors; fumonisins; ochratoxin; trichothecenes; zearalenone; co-contamination by different mycotoxins	Oliveira and Vasconcelos [16]
This review summarized the most predominant types of mycotoxins, the factors affecting their production, and the methods used for their extraction and cleanup from foodstuffs	Types of mycotoxins; factors affecting mycotoxin production; detection of mycotoxins	Elkenany and Awad [13]
This study assessed the presence of aflatoxigenic fungi and mycotoxins in foods, as well as their occurrence, control, and socioeconomic and health implications, from a food safety and quality perspective	Uses of fungi; cultured foods; types of aflatoxigenic fungi; mycotoxins produced by aflatoxigenic fungi; major groups of mycotoxins in foods; health implications of eaten foods contaminated by mycotoxins; economic implications of mycotoxins in foods; prevention and control of mycotoxins in foods	Adeyeye [12]

**Table 2:** Mycotoxin types, along with the foods in which they are mostly found and their respective toxicities.

Mycotoxin	Description	Foods Mostly Found	Toxicities	References
Aflatoxins (aflatoxins B1, B2, G1, G2, M1, M2)	They are produced by several species of *Aspergillus,* especially *Aspergillus parasiticus* and *Aspergillus flavus*, in many commodities	Cereals (wheat, sorghum, rice, acha, millet, guinea corn, corn, etc.), tree nuts (almond, pistachio, coconut, walnut, etc.), oilseeds (peanut, sunflower, cotton seeds, soybean, and sesame), spices (garlic, black pepper, coriander, turmeric, ginger, and chili peppers), etc.	Most aflatoxins are genotoxic, hepatotoxic, mutagenic, etc. and can retard growth in children. AFB1 is most toxic of all and also a very carcinogenic toxin which has been linked directly to many health problems, including liver cancer, in several animals. The understanding of induction of mutations, DNA damage, and metabolism in individuals with dietary exposure to aflatoxins contributes to the general evaluation of their adverse effects on human and animal health. A cross-sectional study conducted on children within the age of 1 to 5 years reported a striking inverse relationship between growth and the level of aflatoxin–albumin adduct.	[2,39]
Ochratoxin A	Ochratoxin A (OTA) is a toxic mycotoxin produced by *A. niger, A. ochraceus*, *Penicillium verrucosum*, and *A. carbonarius*	Cereals (especially wheat and barley) and their products, dry vine fruits, spices, licorice, coffee beans, wine, grape juice, roots, meat, (particularly pork, from animals that consumed infected grains), etc.	OTA is a nephrotoxin and a carcinogen, and it has been directly linked to tumors in the human urinary tract, although the IARC still considers it a possible carcinogen to humans. It is also implicated in various health conditions.	[40,41]
Deoxynivalenol (DON)	DON is a trichothecene mycotoxin produced by fungal species such as *Fusarium graminearum* in cereals	Grains (such as wheat and beans), spices, etc.	DON has been shown to cause acute toxicities in humans, with the main symptom being severe GI toxicity. Consumption of DON-contaminated cereals was linked to several incidents of poisoning in China and at least one outbreak in India.	[42]
Fumonisins (fumonisins B1, B2, B3, B4)	They are mycotoxins produced by *Fusarium* species, including the section Liseola; structurally, they are strongly similar to sphinganine, the sphingolipid backbone precursor; over 15 fumonisins have been described to date	Grains (such as maize, wheat, and beans), spices, etc.	Both fumonisin and *F. verticillioides* contamination in maize positively correlates with cancer of the esophagus in rats, as indicated by studies. Exposure to fumonisins can result in neural tube defects, most likely via disrupting the biosynthesis of sphingolipids and subsequent sphingolipid depletion, which are important for the functions of lipid rafts, particularly folate processing through folate transporters with high affinity.	[43,44,45,46]
Zearalenone (ZEA), also known as F-2 mycotoxin	It is a nonsteroidal estrogenic metabolite produced by some *Fusarium* and *Gibberella* species, such as *Fusarium graminearum*; zearalenone is distributed globally	Grains (especially maize, rice, millet, sorghum, rye, oats, barley, wheat, etc.), spices, etc.	Zearalenone can cause an increase in the incidence of pituitary tumors and liver cell in mice, in line with the hormonal mode of carcinogenic actions. Elevated serum levels of α-zearalenol and ZEA are associated with early puberty. ZEA’s ability to induce hyperkeratotic papilloma in the rat esophageal squamous epithelium forestomach suggests its involvement in tumor development in the gastrointestinal tract. ZEA has been shown to be genotoxic and also has the ability to cause hepatocellular adenomas in mice.	[3,47]
Patulin	It is produced by *P. expansum*, *Penicillium*, *Paecilomyces*, and *Aspergillus* species	Many fruits, vegetables, and grains, especially apple, rotting maize, peanuts, fig, acha, etc.	The acute symptoms of patulin include liver, kidney toxicity, spleen damage and toxicity, and immune toxicity. In humans, gastrointestinal (GI) disturbances, vomiting, and nausea are usually reported. Patulin is genotoxic, but its potential for carcinogenicity is yet to be reported.	[38,42]
Citrinin	It is a mycotoxin first reported in the mold *Penicillium citrinum*; it has been reported in more than 12 *Penicillium* species and numerous *Aspergillus* species	Agricultural crops, such as barley, oats, rye, rice, corn, and wheat, as well foods colored using the *Monascus* pigment	Citrinin is associated with the yellowed rice disease reported in Japan and also acts as nephrotoxin in animal species.	[42]
Ergot alkaloids	The ergot alkaloids are chemical substances released as toxic mixtures of alkaloids in the sclerotia of *Claviceps* species that are known pathogenic microbes of many species of grass	Agricultural crops, such as barley, oats, rye, rice, corn, and wheat	Ergot sclerotia ingestion from infected cereals, commonly in the form of bread made from contaminated flour, results in ergotism, a human disease known as St. Anthony’s fire.	[25]
T-2	T-2 is a trichothecene mycotoxin	Grains (such as maize, rice, millet, sorghum, rye, oats, barley, and wheat), spices, etc.	T-2 has lymphocytic, carcinogenic, cytotoxic, and immunosuppressive actions against mammalian cells. T-2 toxin induced apoptosis and developmental toxicity in zebrafish embryos.	[48,49,50]
Diacetoxyscirpenol (DAS) or 4,15-diacetoxyscirpenol (DAS), also referred to as anguidine	It is a trichothecene mycotoxin secondary metabolite produced by the *Fusarium* genus	Grains (such as wheat, maize, rice, millet, sorghum, soybean, rye, oats, and barley), potato, coffee, etc.	DAS inhibits the production of Ig in the human lymphocytes stimulated by mitogen and can cause esophageal hyperplasia. The major adverse effects following repeated and acute exposure were hematotoxicity and emesis, respectively.	[51,52,53]
Fusarenon X (FusX)	FusX is one of the trichothecenes capable of causing cytotoxicity, carcinogenicity, and immunosuppressive response in animal models and possibly in humans	Oats, cassava, rye, bananas, wheat, maize, rice, millet, sorghum, soybean, mangoes, etc.	In vitro and in vivo, FusX initiates apoptosis in mouse thymocytes, which may be hypothetically applicable to humans. It is very cytotoxic to many cells, and it is believed to have chromosomal effects and to be teratogenic. Fusarenon X has been shown to be carcinogenic, especially to animals.	[54,55,56]
Nivalenol (NIV)	NIV is a trichothecene mycotoxin, which, in nature, is mostly produced by species of *Fusarium*	Cereals and their products, legumes, etc.	Nivalenol, T-2 toxin, and DON were used as bioweapons in some places such as Laos, Cambodia, and Afghanistan, and they were all detected in the vegetation at affected sites, while T-2 toxin was also detected in the blood and urine samples of victims. NIV can increase the rate of induced cancer and mutation, and it is potentially genotoxic. It causes damage in the DNA of colon, jejunum, stomach, bone marrow, and kidney.	[57,58]

**Table 3:** IARC classification of mycotoxins based on their carcinogenicity to humans.

IARC Classification	Mycotoxin (IARC, 2012)	IARC Monograph Reference Year
Group 1: classified as carcinogenic to human	Aflatoxins B1, B2, G1, G2, M1	[84,85,86]
Group 2A: classified as probably carcinogenic to human	Not seen as at the time this study was conducted	
Group 2B: classified as possibly carcinogenic to human	Ochratoxin A, fumonisin B1, fumonisin B2, fusarin C, sterigmatocystin	[71,87,88,89,90]
Group 3: not classifiable as carcinogenic to human	Deoxynivalenol, patulin, citrinin, zearalenone, fusarenone X	[89,91]
Group 4: probably not carcinogenic to human	Not seen as at the time this study was conducted	

## Data Availability

Not applicable.

## References

[1] Turner N.W., Subrahmanyam S., Piletsky S.A. (2009). Analytical methods for the determination of mycotoxins: A review. Anal. Chim. Acta.

[2] WHO (2018). Mycotoxins. https://www.who.int/news-room/fact-sheets/detail/mycotoxins.

[3] Bulgaru C.V., Marin D.E., Pistol G.C., Taranu I. (2021). Zearalenone and the Immune Response. Toxins.

[4] Claeys L., Romano C., De Ruyck K., Wilson H., Fervers B., Korenjak M., Zavadil J., Gunter M.J., De Saeger S., De Boevre M. (2020). Mycotoxin exposure and human cancer risk: A systematic review of epidemiological studies. Compr. Rev. Food Sci. Food Saf..

[5] Awuchi C.G., Nwozo S., Salihu M., Odongo G.A., Sarvarian M., Okpala C.O.R. (2022). Mycotoxins’ Toxicities—From Consumer Health Safety Concerns, to Mitigation/Treatment Strategies: A Perspective Review. J. Chem. Health Risks.

[6] Cornely A.O. (2008). Aspergillus to Zygomycetes: The causes, risk factors, prevention, & treatment of invasive fungal infections. Infection.

[7] Schaafsma A.W., Hooker C.D. (2007). Climatic models to predict the occurrence of Fusarium toxins in wheat & maize. Int. J. Food Microbiol..

[8] Zhou Q., Tang D. (2020). Recent advances in photoelectrochemical biosensors for analysis of mycotoxins in food. TrAC Trends Anal. Chem..

[9] Fox E.M., Howlett B.J. (2008). Secondary metabolism: Regulation and role in fungal biology. Curr. Opin. Microbiol..

[10] Chen A., Mao X., Sun Q., Wei Z., Li J., You Y., Zhao J., Jiang G., Wu Y., Wang L. (2021). Alternaria Mycotoxins: An Overview of Toxicity, Metabolism, and Analysis in Food. J. Agric. Food Chem..

[11] Iqbal S.Z. (2021). Mycotoxins in food, recent development in food analysis and future challenges; a review. Curr. Opin. Food Sci..

[12] Adeyeye S.A.O. (2020). Aflatoxigenic fungi and mycotoxins in food: A review. Crit. Rev. Food Sci. Nutr..

[13] Elkenany R.M., Awad A. (2020). Types of Mycotoxins and different approaches used for their detection in foodstuffs. Mansoura Vet. Med. J..

[14] Pierzgalski A., Bryła M., Kanabus J., Modrzewska M., Podolska G. (2021). Updated Review of the Toxicity of Selected *Fusarium* Toxins and Their Modified Forms. Toxins.

[15] Lu Q., Qin J.-A., Fu Y.-W., Luo J.-Y., Lu J.-H., Logrieco A.F., Yang M.-H. (2020). Modified mycotoxins in foodstuffs, animal feed, and herbal medicine: A systematic review on global occurrence, transformation mechanism and analysis methods. TrAC Trends Anal. Chem..

[16] Oliveira M., Vasconcelos V. (2020). Occurrence of Mycotoxins in Fish Feed and Its Effects: A Review. Toxins.

[17] Ráduly Z., Szabó L., Madar A., Pócsi I., Csernoch L. (2020). Toxicological and Medical Aspects of Aspergillus-Derived Mycotoxins Entering the Feed and Food Chain. Front. Microbiol..

[18] Awuchi C.G., Ondari E.N., Ogbonna C.U., Upadhyay A.K., Baran K., Okpala C.O.R., Korzeniowska M., Guiné R.P.F. (2021). Mycotoxins Affecting Animals, Foods, Humans and Plants: Types, Occurrence, Toxicities, Action Mechanisms, Prevention and Detoxification Strategies—A Revisit. Foods.

[19] Hussein H.S., Brasel M.J. (2001). Toxicity, metabolism, & impact of mycotoxins on humans & animals. Toxicology.

[20] Khaneghah A.M., Fakhri Y., Gahruie H.H., Niakousari M., Sant’Ana A.S. (2019). Mycotoxins in cereal-based products during 24 years (1983–2017): A global systematic review. Trends Food Sci. Technol..

[21] Holanda D.M., Kim S.W. (2021). Mycotoxin Occurrence, Toxicity, and Detoxifying Agents in Pig Production with an Emphasis on Deoxynivalenol. Toxins.

[22] Keller N.P., Turner G., Bennett W.J. (2005). Fungal secondary metabolism—From the biochemistry to the genomics. Nat. Rev. Microbiol..

[23] Melina R. (2020). Sex-Change Chicken: Gertie d Hen Becomes Bertie d Cockerel.

[24] Boonen J., Malysheva S., Taevernier L., Diana Di M.J., De Saeger S., De Spiegeleer B. (2012). The human skin penetrations of selected model of mycotoxins. Toxicology.

[25] Bennett J.W., Klich M. (2003). Mycotoxins. Clin. Microbiol Rev..

[26] Ashiq S., Hussain M., Ahmad B. (2014). The natural occurrence of mycotoxin in medicinal plants: Review. Fungal Genet. Biol..

[27] Do K.H., An J.T., Oh S.K., Moon Y. (2015). Nation-Based Occurrence & Endogenous Biological Reduction of Mycotoxins in Medicinal Herbs & Spices. Toxins.

[28] Agriopoulou S., Stamatelopoulou E., Varzakas T. (2020). Advances in Occurrence, Importance, and Mycotoxin Control Strategies: Prevention and Detoxification in Foods. Foods.

[29] Pestka J.J., Yike I., Dearborn G.D., Ward M.D., Harkema J.R. (2008). Stachybotrys chartarum, trichothecene mycotoxins, & damp building-related illness: New insights into public health enigma. Toxicol. Sci..

[30] Susan S.L. (2006). Dog Keep Dying: Too Many Owners Remain Unaware of the Toxic Dog Food.

[31] Veprikova Z., Zachariasova M., Dzuman Z., Zachariasova A., Fenclova M., Slavikova P.M., Vaclavikova M., Mastovska K., Hengst D., Hajslova J.K. (2015). Mycotoxins in Plant-Based Dietary Supplement: Hidden Health Risks for Consumers. J. Agric. Food Chem..

[32] Bullerman L., Bianchini A. (2007). Stability of mycotoxin during food processing. Int. J. Food Microbiol..

[33] Kabak B., Dobson A.D., Var I. (2006). The strategies to prevent mycotoxin contamination of food & animal feed: A review. Crit. Rev. Food Sci. Nutr..

[34] Lewis L., Mary O., Henry N., Helen S.R., George L., Stephanie K., Jack N., Lorraine B., Abdikher M.D., Ambrose M. (2005). Aflatoxin contamination of commercial maize products during an outbreak of acute aflatoxicosis in eastern and central Kenya. Environ. Health Perspect..

[35] Muñoz K., Wagner M., Pauli F., Christ J., Reese G. (2021). Knowledge and Behavioral Habits to Reduce Mycotoxin Dietary Exposure at Household Level in a Cohort of German University Students. Toxins.

[36] Jeswal P., Kumar D. (2015). Mycobiota and Natural Incidence of Aflatoxin, Ochratoxin A, and Citrinin in Indian Spices Confirmed by LC-MS/MS. Int. J. Microbiol..

[37] Desjardins A.E., Proctor H.R. (2007). Molecular biology of Fusarium mycotoxins. Int. J. Food Microbiol..

[38] Chinaza G.A., Clifford I.O., Chika C.O., Victory S.I. (2019). Evaluation of Patulin Levels and impacts on the Physical Characteristics of Grains. Int. J. Adv. Acad. Res..

[39] Lombard M.J. (2014). Mycotoxins exposure and infant and young child growth in Africa: What do we know?. Ann. Nutr. Metab..

[40] Bayman P., Baker L.J. (2006). Ochratoxins: A global perspective. Mycopathologia.

[41] Mateo R., Medina A., Mateo M.E., Mateo F., Jiménez M. (2007). An overview of ochratoxin A in beer & wine. Int. J. Food Microbiol..

[42] Adam M.A.A., Tabana Y.M., Musa K.B., Sandai D.A. (2017). Effects of different mycotoxins on humans, cell genome and their involvement in cancer (Review). Oncol. Rep..

[43] Marasas W.F.O., Miller J.D., Riley R.T., Visconti A. (2000). Environmental Health Criteria 219: Fumonisin B1.

[44] Gelineau-van Waes J., Starr L., Maddox J.R., Aleman F., Voss K.A., Wilberding J., Riley R.T. (2005). Maternal fumonisin exposure and risk for neural tube defects: Disruption of sphingolipid metabolism and folate transport in an in vivo mouse model. Birth Defects Res. A Clin. Mol. Teratol..

[45] Sun G., Wang S., Hu X., Su J., Huang T., You J., Tang L., Gao W., Wang J.-S. (2007). Fumonisin B1 contamination of home-grown corn in high-risk areas for esophageal and liver cancer in China. Food Addit. Contam..

[46] Renaud J.B.R., Kelman M.J., Qi T.F., Seifert K.A., Sumarah M.W. (2015). Product ion filtering with rapid polarity switching for the detection of all fumonisins and AAL-toxins. Rapid Commun. Mass Spectrom..

[47] Rogowska A., Pomastowski P., Sagandykova G., Buszewski B. (2019). Zearalenone and its metabolites: Effect on human health, metabolism and neutralisation methods. Toxicon.

[48] Yuan G., Wang Y., Yuan X., Zhang T., Zhao J., Huang L., Peng S. (2014). T-2 toxin induces developmental toxicity and apoptosis in zebrafish embryos. J. Environ. Sci..

[49] Wan Q., Wu G., He Q., Tang H., Wang Y. (2015). The toxicity of acute exposure to T-2 toxin evaluated by the metabonomics technique. Mol. BioSystems.

[50] Adhikari M., Negi B., Kaushik N., Adhikari A., Al-Khedhairy A.A., Kaushik N.K., Choi E.H. (2017). T-2 mycotoxin: Toxicological effects and decontamination strategies. Oncotarget.

[51] Knutsen H.K., Alexander J., Barregård L., Bignami M., Brüschweiler B., Ceccatelli S., Cottrill B., Dinovi M., EFSA CONTAM Panel (EFSA Panel on Contaminants in the Food Chain) (2018). Scientific Opinion on the risk to human and animal health related to the presence of 4,15-diacetoxyscirpenol in food and feed. EFSA J..

[52] Wu Q., Kuca K., Nepovimova E., Wu W. (2020). Type A Trichothecene Diacetoxyscirpenol-Induced Emesis Corresponds to Secretion of Peptide YY and Serotonin in Mink. Toxins.

[53] Daud N., Currie V., Duncan G., Farquharson F., Yoshinari T., Louis P., Gratz S.W. (2020). Prevalent Human Gut Bacteria Hydrolyse and Metabolise Important Food-Derived Mycotoxins and Masked Mycotoxins. Toxins.

[54] Wijnands L.M., van Leusden F.M. (2000). An Overview of Adverse Health Effects Caused by Mycotoxins and Bioassays for Their Detection; Research for Man and Environment, RIVM Report 257852,004; Rijksinstituut voor Volksgezondheid en Milieu RIVM. http://rivm.openrepository.com/rivm/bitstream/10029/9410/1/257852004.pdf.

[55] Bony S., Olivier-Loiseau L., Carcelen M., Devaux A. (2007). Genotoxic potential associated with low levels of the Fusarium mycotoxins nivalenol and fusarenon x in a human intestinal cell line. Toxicol Vitr..

[56] Sabharwal A., Bibekananda K., Santiago R.C., Holmberg S.R., Kendall B.L., Cotter R.P., WareJoncas Z., Clark K.J., Ekker S.C. The FusX TALE Base Editor (FusXTBE) for rapid mitochondrial DNA programming of human cells in vitro and zebrafish disease models in vivo. bioRxiv.

[57] Scientific Committee on Food (2000). Opinion of the Scientific Committee on Food on Fusarium Toxins Part 41: Nivalenol.

[58] Gupta R.C. (2015). Handbook of Toxicology of Chemical Warfare Agents.

[59] Martins M.L., Martins M.H., Bernardo F. (2001). Aflatoxins in spices marketed in Portugal. Food Addit. Contam..

[60] Awuchi C.G., Amagwula I.O., Priya P., Kumar R., Yezdani U., Khan M.G. (2020). Aflatoxins in Foods and Feeds: A Review On Health Implications, Detection, And Control. Bull. Environ. Pharmacol. Life Sci..

[61] Wild C.P., Turner P.C. (2002). The toxicology of aflatoxins as a basis for public health decisions. Mutagenesis.

[62] Urusov A.E., Zherdev A.V., Petrakova A.V., Sadykhov E.G., Koroleva O.V., Dzantiev B.B. (2015). Rapid multiple immunoenzyme assay of mycotoxins. Toxins.

[63] Anfossi L., Giovannoli C., Baggiani C. (2016). Mycotoxin detection. Curr. Opin. Biotechnol..

[64] Qiu J., Dong F., Yu M., Xu J., Shi J. (2016). Effect of preceding crop on *Fusarium* species and mycotoxin contamination of wheat grains. J. Sci. Food Agric..

[65] Thelen K., Dressman J.B. (2009). Cytochrome P450-mediated metabolism in the human gut wall. J. Pharm. Pharm..

[66] Kamdem L.K., Meineke I., Gödtel-Armbrust U., Brockmöller J., Wojnowski L. (2006). Dominant contribution of P450 3A4 to the hepatic carcinogenic activation of aflatoxin B. Chem. Res. Toxicol..

[67] Awuchi C.G., Owuamanam I.C., Ogueke C.C., Hannington T. (2020). The Impacts of Mycotoxins on the Proximate Composition and Functional Properties of Grains. Eur. Acad. Res..

[68] Wojnowski L., Turner P.C., Pedersen B., Hustert E., Brockmöller J., Mendy M., Whittle H.C., Kirk G., Wild C.P. (2004). Increased levels of aflatoxin-albumin adducts are associated with CYP3A5 polymorphisms in The Gambia, West Africa. Pharmacogenetics.

[69] Turner P.C., Collinson A.C., Cheung Y.B., Gong Y.Y., Hall A.J., Prentice A.M., Wild C.P. (2007). Aflatoxin exposure in utero causes growth faltering in Gambian infants. Int. J. Epidemiol..

[70] Hussain S.P., Schwank J., Staib F., Wang X.W., Harris C.C. (2007). TP53 mutations and hepatocellular carcinoma: Insights into the etiology and pathogenesis of liver cancer. Oncogene.

[71] IARC Working Group on the Evaluation of Carcinogenic Risks to Humans (2002). IARC Monographs on the Evaluation of Carcinogenic Risks to Humans.

[72] Wild C.P., Gong Y.Y. (2010). Mycotoxins and human disease: A largely ignored global health issue. Carcinogenesis.

[73] Groopman J.D., Kensler T.W., Wild C.P. (2008). Protective interventions to prevent aflatoxin-induced carcinogenesis in developing countries. Annu. Rev. Public Health.

[74] Guengerich F.P., Johnson W.W., Shimada T., Ueng Y.-F., Yamazaki H., Langouët S. (1998). Activation and detoxication of aflatoxin B. Mutat. Res..

[75] Johnson D.N., Egner P.A., Obrian G., Glassbrook N., Roebuck B.D., Sutter T.R., Payne G.A., Kensler T.W., Groopman J.D. (2008). Quantification of urinary aflatoxin B1 dialdehyde metabolites formed by aflatoxin aldehyde reductase using isotope dilution tandem mass spectrometry. Chem. Res. Toxicol..

[76] Hall A.J., Wild C.P., Eaton D.L., Groopman J.D. (1994). Epidemiology of aflatoxin-related disease. The Toxicology of Aflatoxins: Human Health, Veterinary, and Agricultural Significance.

[77] Azziz-Baumgartner E., Lindblade K., Gieseker K., Rogers H.S., Kieszak S., Njapau H., Schleicher R., McCoy L.F., Misore A., DeCock K. (2005). Case-control study of an acute aflatoxicosis outbreak, Kenya, 2004. Environ. Health Perspect.

[78] McCoy L.F., Scholl P.F., Sutcliffe A.E., Kieszak S.M., Powers C.D., Rogers H.S., Gong Y.Y., Groopman J.D., Wild C.P., Schleicher R.L. (2008). Human aflatoxin albumin adducts quantitatively compared by ELISA, HPLC with fluorescence detection, and HPLC with isotope dilution mass spectrometry. Cancer Epidemiol. Biomark. Prev..

[79] Wang L.Y., Hatch M., Chen C.J., Levin B., You S.-L., Lu S.-N., Wu M.-H., Wu W.-P., Wang L.-W., Wang Q. (1996). Aflatoxin exposure and risk of hepatocellular carcinoma in Taiwan. Int. J. Cancer.

[80] Wu H.C., Wang Q., Yang H.I., Ahsan H., Tsai W.-Y., Wang L.-Y., Chen S.-Y., Chen C.-J., Santella R.M. (2009). Aflatoxin Bexposure, hepatitis B virus infection, and hepatocellular carcinoma in Taiwan. Cancer Epidemiol. Biomark. Prev..

[81] Kirk G.D., Lesi O.A., Mendy M., Szymañska K., Whittle H., Goedert J.J., Hainaut P., Montesano R. (2005). 249(ser) TP53 mutation in plasma DNA, hepatitis B viral infection, and risk of hepatocellular carcinoma. Oncogene.

[82] Omer R.E., Kuijsten A., Kadaru A.M., Kok F.J., Idris M.O., El Khidir I.M., van’t Veer P. (2004). Population-attributable risk of dietary aflatoxins and hepatitis B virus infection with respect to hepatocellular carcinoma. Nutr. Cancer.

[83] Kuniholm M.H., Lesi O.A., Mendy M., Akano A.O., Sam O., Hall A.J., Whittle H., Bah E., Goedert J.J., Hainaut P. (2008). Aflatoxin exposure and viral hepatitis in the etiology of liver cirrhosis in The Gambia, West Africa. Environ. Health Perspect..

[84] IARC (2006). IARC Monographs on the Evaluation of Carcinogenic Risks to Humans. Preamble. https://monographs.iarc.fr/wp-content/uploads/2018/06/CurrentPreamble.pdf.

[85] IARC (2012). Agents classified by the IARC monographs, volumes 1–104. IARC Monogr..

[86] IARC (2019). Agents Classified by the IARC Monographs. Volumes 1–123. https://monographs.iarc.fr/agents-classified-by-the-iarc/.

[87] IARC (1987). IARC Monographs on the Evaluation of Carcinogenic Risks to Humans. https://monographs.iarc.fr/iarc-monographs-on-the-evaluation-of-carcinogenic-risks-to-humans-80/.

[88] IARC (2019). Report of the Advisory Group to Recommend Priorities for IARC Monographs during 2020–2024. IARC Monographs on the Evaluation of Carcinogenic Risks to Humans.

[89] IARC (1993). IARC Monographs on the Evaluation of Carcinogenic Risks to Humans. https://monographs.iarc.fr/iarc-monographs-on-the-evaluation-of-carcinogenic-risks-to-humans-65/.

[90] IARC (1993). IARC Monographs on the Evaluation of Carcinogenic Risks to Humans.

[91] IARC (1987). IARC Monographs on the Evaluation of the Carcinogenic Risk of Chemicals to Humans.

[92] WHO (2002). Evaluation of Certain Mycotoxins in Food: Fifty-Sixth Report of the Joint FAO/WHO Expert Committee on Food Additives.

[93] Williams J.H., Phillips T.D., Jolly P.E., Stiles J.K., Jolly C.M., Aggarwal D. (2004). Human aflatoxicosis in developing countries: A review of toxicology, exposure, potential health consequences, and interventions. Am. J. Clin. Nutr..

[94] Turner P.C., Moore S.E., Hall A.J., Prentice A.M., Wild C.P. (2003). Modification of immune function through exposure to dietary aflatoxin in Gambian children. Environ. Health Perspect..

[95] Jiang Y., Jolly P.E., Ellis W.O., Wang J.-S., Phillips T.D., Williams J.H. (2005). Aflatoxin Balbumin adduct levels and cellular immune status in Ghanaians. Int. Immunol..

[96] Jiang Y., Jolly P.E., Preko P., Wang J.-S., Ellis W.O., Phillips T.D., Williams J.H. (2008). Aflatoxin-related immune dysfunction in health and in human immunodeficiency virus disease. Clin. Dev. Immunol..

[97] Hendrickse R.G., Coulter J.B., Lamplugh S.M., Macfarlane S.B., Williams T.E., Omer M.I., Suliman G.I. (1982). Aflatoxins and kwashiorkor: A study in Sudanese children. Br. Med. J. (Clin. Res. Ed.).

[98] De Vries H.R., Maxwell S.M., Hendrickse R.G. (1989). Foetal and neonatal exposure to aflatoxins. Acta Paediatr. Scand..

[99] Okoth S.A., Ohingo M. (2004). Dietary aflatoxin exposure and impaired growth in young children from Kisumu District, Kenya: Cross sectional study. Afr. JHealth Sci..

[100] Gong Y.Y., Hounsa A., Egal S., Turner P.C., Sutcliffe A.E., Hall A.J., Cardwell K., Wild C.P. (2004). Postweaning exposure to aflatoxin results in impaired child growth: A longitudinal study in Benin, West Africa. Environ. Health Perspect..

[101] Black R.E., Morris S.S., Bryce J. (2003). Where and why are 10 million children dying every year?. Lancet.

[102] WHO (2008). Safety Evaluation of Certain Food Additives and Contaminants: Prepared by the Sixty-Eighth Meeting of the Joint FAO/WHO Expert Committee on Food Additives (JECFA).

[103] Studer-Rohr I., Schlatter J., Dietrich D.R. (2000). Kinetic parameters and intraindividual fluctuations of ochratoxin A plasma levels in humans. Arch. Toxicol..

[104] Marin-Kuan M., Cavin C., Delatour T., Schilter B. (2008). Ochratoxin A carcinogenicity involves a complex network of epigenetic mechanisms. Toxicon.

[105] Pfohl-Leszkowicz A., Manderville R.A. (2007). Ochratoxin A: An overview on toxicity and carcinogenicity in animals and humans. Mol. Nutr. Food Res..

[106] Mally A., Dekant W. (2009). Mycotoxins and the kidney: Modes of action for renal tumor formation by ochratoxin A in rodents. Mol. Nutr. Food Res..

[107] Mantle P.G., Faucet-Marquis V., Manderville R.A., Squillaci B., Pfohl-Leszkowicz A. (2010). Structures of covalent adducts between DNA and ochratoxin A: A new factor in debate about genotoxicity and human risk assessment. Chem. Res. Toxicol..

[108] Dietrich D.R., Heussner A.H., O’Brien E. (2005). Ochratoxin A: Comparative pharmacokinetics and toxicological implications (experimental and domestic animals and humans). Food Addit. Contam..

[109] Bow D.A., Perry J.L., Simon J.D., Pritchard J.B. (2006). The impact of plasma protein binding on the renal transport of organic anions. J. Pharm. Exp..

[110] Adler M., Müller K., Rached E., Dekant W., Mally A. (2009). Modulation of key regulators of mitosis linked to chromosomal instability is an early event in ochratoxin A carcinogenicity. Carcinogenesis.

[111] Arbillaga L., Vettorazzi A., Gil A.G., van Delft J.H.M., García-Jalón J.A., López de Cerain A. (2008). Gene expression changes induced by ochratoxin A in renal and hepatic tissues of male F344 rat after oral repeated administration. Toxicol. Appl. Pharm..

[112] Cavin C., Delatour T., Marin-Kuan M., Fenaille F., Holzhäuser D., Guignard G., Bezençon C., Piguet D., Parisod V., Richoz-Payot J. (2009). Ochratoxin A-mediated DNA and protein damage: Roles of nitrosative and oxidative stresses. Toxicol. Sci..

[113] Mayer S., Curtui V., Usleber E., Gareis M. (2007). Airborne mycotoxins in dust from grain elevators. Mycotoxin Res..

[114] WHO (2011). Safety Evaluation of Certain Contaminants in Food: Prepared by the Seventy-Second Meeting of the Joint FAO/WHO Expert Committee on Food Additives (JECFA).

[115] Snijders C.H.A., Miller J.D., Trenholm H.L. (1994). Breeding for resistance to Fusarium diseases in wheat and maize. Mycotoxins in Grain: Compounds Other than Aflatoxin.

[116] Oufensou S., Dessì A., Dallocchio R., Balmas V., Azara E., Carta P., Migheli Q., Delogu G. (2021). Molecular Docking and Comparative Inhibitory Efficacy of Naturally Occurring Compounds on Vegetative Growth and Deoxynivalenol Biosynthesis in *Fusarium culmorum*. Toxins.

[117] Pestka J.J. (2008). Mechanisms of deoxynivalenol-induced gene expression and apoptosis. Food Addit. Contam. Part A Chem. Anal. Control Expo. Risk Assess..

[118] Pass C., MacRae V.E., Ahmed S.F., Farquharson C. (2009). Inflammatory cytokines and the GH/IGF-I axis: Novel actions on bone growth. Cell Biochem. Funct..

[119] Amuzie C.J., Pestka J.J. (2010). Suppression of insulin-like growth factor acid-labile subunit expression–a novel mechanism for deoxynivalenol-induced growth retardation. Toxicol. Sci..

[120] Pestka J.J., Smolinski A.T. (2005). Deoxynivalenol: Toxicology and potential effects on humans. J. Toxicol. Environ. Health B Crit. Rev..

[121] Gray J.S., Pestka J.J. (2007). Transcriptional regulation of deoxynivalenol-induced IL-8 expression in human monocytes. Toxicol. Sci..

[122] Ehrlich V., Darroudi F., Uhl M., Steinkellner H., Zsivkovits M., Knasmueller S. (2002). Fumonisin B1 is genotoxic in human derived hepatoma (HepG2) cells. Mutagenesis.

[123] Illueca F., Vila-Donat P., Calpe J., Luz C., Meca G., Quiles J.M. (2021). Antifungal Activity of Biocontrol Agents In Vitro and Potential Application to Reduce Mycotoxins (Aflatoxin B1 and Ochratoxin A). Toxins.

[124] Domijan A.M., Zeljezić D., Kopjar N., Peraica M. (2006). Standard and Fpg-modified comet assay in kidney cells of ochratoxin A- and fumonisin B-treated rats. Toxicology.

[125] Stockmann-Juvala H., Savolainen K. (2008). A review of the toxic effects and mechanisms of action of fumonisin B. Hum. Exp. Toxicol..

[126] Gelderblom W.C.A., Marasas W.F.O., Lebepe-Mazur S., Swanevelder S., Abel S. (2008). Cancer initiating properties of fumonisin Bin a short-term rat liver carcinogenesis assay. Toxicology.

[127] Desai K., Sullards M.C., Allegood J., Wang E., Schmelz E.M., Hartl M., Humpfd H.-U., Liottae D.C., Peng Q., Merrill A.H. (2002). Fumonisins and fumonisin analogs as inhibitors of ceramide synthase and inducers of apoptosis. Biochim. Biophys. Acta (BBA)—Mol. Cell Biol. Lipids.

[128] Sharma R.P., Riley R.T., Voss K.A., DeKoe W.J., Samson R.A., van Egmond H.P., Gilbert J., Sabino M. (2000). Cytokine involvement in fumonisin-induced cellular toxicity. Mycotoxins and Phytoxins in Perspective at the Turn of the Millenium.

[129] Gelderblom W.C.A., Riedel S., Burger H.-M., Abel S., Marasas W.F.O., Sianta D.P., Trucksess M.W., Scott P.M., Herman E.M. (2008). Carcinogenesis by the fumonisins: Mechanisms, risk analyses, and implications. Food Contaminants: Mycotoxins and Food Allergens.

[130] Taranu I., Marin D.E., Bouhet S., Pascale F., Bailly J.-D., Miller J.D., Pinton P., Oswald I.P. (2005). Mycotoxin fumonisin B1 alters the cytokine profile and decreases the vaccinal antibody titer in pigs. Toxicol. Sci..

[131] Marasas W.F. (2001). Discovery and occurrence of the fumonisins: A historical perspective. Environ. Health Perspect..

[132] Qiu M.F., Liu X.M. (2001). Determination of sphinganine, sphingosine and Sa/So ratio in urine of humans exposed to dietary fumonisin B. Food Addit. Contam..

[133] Kozieł M.J., Ziaja M., Piastowska-Ciesielska A.W. (2021). Intestinal Barrier, Claudins and Mycotoxins. Toxins.

[134] Nikièma P.N., Worrillow L., Traoré A.S., Wild C.P., Turner P.C. (2004). Fumonisin contamination of maize in Burkina Faso, West Africa. Food Addit. Contam..

[135] Xu L., Cai Q., Tang L., Wang S., Hu X., Su J., Sun G., Wang J.-S. (2010). Evaluation of fumonisin biomarkers in a cross-sectional study with two high-risk populations in China. Food Addit. Contam. Part A Chem. Anal. Control Expo. Risk Assess..

[136] Van der Westhuizen L., Shephard G.S., Rheeder J.P., Burger H.-M. (2010). Individual fumonisin exposure and sphingoid base levels in rural populations consuming maize in South Africa. Food Chem. Toxicol..

[137] Gelderblom W.C., Marasas W.F., Lebepe-Mazur S., Swanevelder S., Vessey C.J., Hall P.d.l.M. (2002). Interaction of fumonisin B1 and aflatoxin B1 in a short-term carcinogenesis model in rat liver. Toxicology.

[138] Carlson D.B., Williams D.E., Spitsbergen J.M., Ross P.F., Bacon C.W., Meredith F.I., Riley R.T. (2001). Fumonisin B1 promotes aflatoxin B1 and N-methyl-N′-nitro-nitrosoguanidine-initiated liver tumors in rainbow trout. Toxicol. Appl. Pharm..

[139] Li F.-Q., Yoshizawa T., Kawamura O., Luo X.-Y., Li Y.-W. (2001). Aflatoxins and fumonisins in corn from the high-incidence area for human hepatocellular carcinoma in Guangxi, China. J. Agric. Food Chem..

[140] Sadler T.W., Merrill A.H., Stevens V.L., Sullards M.C., Wang E., Wang P. (2002). Prevention of fumonisin B1-induced neural tube defects by folic acid. Teratology.

[141] Marasas W.F.O., Riley R.T., Hendricks K.A., Stevens V.L., Sadler T.W., Waes J.G., Missmer S.A., Cabrera J., Torres O., Gelderblom W.C.A. (2004). Fumonisins disrupt sphingolipid metabolism, folate transport, and neural tube development in embryo culture and in vivo: A potential risk factor for human neural tube defects among populations consuming fumonisin-contaminated maize. J. Nutr..

[142] Gelineau-van Waes J., Voss K.A., Stevens V.L., Speer M.C., Riley R.T. (2009). Maternal fumonisin exposure as a risk factor for neural tube defects. Adv. Food Nutr. Res..

[143] Zinedine A., Soriano J.M., Moltó J.C., Mañes J. (2007). Review on the toxicity, occurrence, metabolism, detoxification, regulations and intake of zearalenone: An oestrogenic mycotoxin. Food Chem. Toxicol..

[144] Wang N., Wu W., Pan J., Long M. (2019). Detoxification Strategies for Zearalenone Using Microorganisms: A Review. Microorganisms.

[145] WHO (2001). Safety Evaluation of Certain Mycotoxins in Food: Prepared by the Fifty-Sixth Meeting of the Joint FAO/WHO Expert Committee on Food Additives (JECFA).

[146] Zheng W., Feng N., Wang Y., Noll L., Xu S., Liu X., Lu N., Zou H., Gu J., Yuan Y. (2019). Effects of zearalenone and its derivatives on the synthesis and secretion of mammalian sex steroid hormones: A review. Food Chem. Toxicol..

[147] Fink-Gremmels J., Malekinejad H. (2007). Clinical effects and biochemical mechanisms associated with exposure to the mycoestrogen zearalenone. Anim. Feed Sci. Technol..

[148] Ding X., Lichti K., Staudinger J.L. (2006). The mycoestrogen zearalenone induces CYP3A through activation of the pregnane X receptor. Toxicol. Sci..

[149] Rai A., Das M., Tripathi A. (2020). Occurrence and toxicity of a fusarium mycotoxin, zearalenone. Crit. Rev. Food Sci. Nutr..

[150] Richard J.L. (2007). Some major mycotoxins and their mycotoxicoses—An overview. Int. J. Food Microbiol..

[151] Ouanes Z., Abid S., Ayed I., Anane R., Mobio T., Creppy E.E., Bacha H. (2003). Induction of micronuclei by Zearalenone in vero monkey kidney cells and in bone marrow cells of mice: Protective effect of Vitamin E. Mutat. Res..

[152] Schoental R. (1974). Letter: Role of podophyllotoxin in the bedding and dietary zearalenone on incidence of spontaneous tumors in laboratory animals. Cancer Res..

[153] Tkaczyk A., Jedziniak P., Zielonka Ł., Dąbrowski M., Ochodzki P., Rudawska A. (2021). Biomarkers of Deoxynivalenol, Citrinin, Ochratoxin A and Zearalenone in Pigs after Exposure to Naturally Contaminated Feed Close to Guidance Values. Toxins.

[154] Pfohl-Leszkowicz A., Chekir-Ghedira L., Bacha H. (1995). Genotoxicity of zearalenone, an estrogenic mycotoxin: DNA adduct formation in female mouse tissues. Carcinogenesis.

[155] Moss O.M. (2008). “fungi”, quality & safety issues in fresh fruits & vegetables. J. Appl. Microbiol..

[156] Schothorst R.C., van Egmond H.P. (2004). Report from SCOOP task 3.2.10 ‘collection of occurrence data of Fusarium toxins in food and assessment of dietary intake by the population of Eu member states’. Subtask: Trichothecenes. Subtask: Trichothecenes. Toxicol. Lett..

[157] Trucksess M.W., Scott M.P. (2008). Mycotoxins in botanicals & dried fruits: A review. Food Addit. Contam..

[158] Schumacher D.M., Metzler M., Lehmann L. (2005). Mutagenicity of the mycotoxin patulin in cultured Chinese hamster V79 cells, and its modulation by intracellular glutathione. Arch. Toxicol..

[159] Mahfoud R., Maresca M., Garmy N., Fantini J. (2002). The mycotoxin patulin alters the barrier function of the intestinal epithelium: Mechanism of action of the toxin and protective effects of glutathione. Toxicol. Appl. Pharm..

[160] Matossian M.K. (2010). Poisons of the Past: Molds, Epidemics, and History. Yale University Press, New Haven and London, 1989. Arch. Nat. Hist..

[161] Bhat R., Rai R.V., Karim A.A. (2010). Mycotoxins in food and feed: Present status and future concerns. Compr. Rev. Food Sci. Food Saf..

[162] Wojtacha P., Trybowski W., Podlasz P., Żmigrodzka M., Tyburski J., Polak-Śliwińska M., Jakimiuk E., Bakuła T., Baranowski M., Żuk-Gołaszewska K. (2021). Effects of a Low Dose of T-2 Toxin on the Percentage of T and B Lymphocytes and Cytokine Secretion in the Porcine Ileal Wall. Toxins.

[163] Mackei M., Orbán K., Molnár A., Pál L., Dublecz K., Husvéth F., Neogrády Z., Mátis G. (2020). Cellular Effects of T-2 Toxin on Primary Hepatic Cell Culture Models of Chickens. Toxins.

[164] Liu Y., Wang H., Zhang M., Wang J., Zhang Z., Wang Y., Sun Y., Zhang Z. (2021). Protective effect of selenomethionine on T-2 toxin-induced liver injury in New Zealand rabbits. BMC Vet. Res..

[165] Liu Y., Yang Y., Dong R., Zhang Z., Jia F., Yu H., Wang Y., Zhang Z. (2020). Protective effect of selenomethionine on intestinal injury induced by T-2 toxin. Res. Vet. Sci..

[166] Gupta R.C. (2018). Veterinary Toxicology: Basic and Clinical Principles.

[167] Ma A.C., McNulty M.S., Poshusta T.L., Campbell J.M., Martínez-Gálvez G., Argue D.P., Lee H.B., Urban M.D., Bullard C.E., Blackburn P.R. (2016). FusX: A Rapid One-Step Transcription Activator-Like Effector Assembly System for Genome Science. Hum. Gene Ther..

[168] Deshmaneand S.L., Kremlev S., Amini S., Sawaya B.E. (2009). Monocyte Chemoattractant Protein-1 (MCP-1): An Overview. J. Interferon Cytokine Res..

[169] Nagashima H., Nakagawa H., Kushiro M. (2012). Environ Toxicol Pharmacol. Environ. Toxicol. Pharmacol..

[170] Minervini F., Fornelli F., Flynn K.M. (2004). Toxicity and apoptosis induced by the mycotoxins nivalenol, deoxynivalenol and fumonisin B1 in a human erythroleukemia cell line. Toxicol. Vitr..

[171] Taranu I., Marin D.E., Burlacu R., Pinton P., Damian V., Oswald I.P. (2010). Comparative aspects of in vitro proliferation of human and porcine lymphocytes exposed to mycotoxins. Arch. Anim. Nutr..

[172] Pillay D., Chuturgoon A.A., Nevines E., Manickum T., Deppe W., Dutton M.F. (2002). The quantitative analysis of zearalenone and its derivatives in plasma of patients with breast and cervical cancer. Clin. Chem. Lab. Med..

[173] Belhassen H., Jimenez-Diaz I., Arrebola J.P., Ghali R., Ghorbel H., Olea N., Hedili A. (2015). Zearalenone and its metabolites in urine and breast cancer risk: A case–control study in Tunisia. Chemosphere.

[174] Stephany R.W. (2009). Hormonal growth promoting agents in food producing animals. Handbook of Experimental Pharmacology.

[175] Knutsen H.K., Barregård L., Bignami M., Brüschweiler B., Ceccatelli S., Cottrill B., Dinovi M., Edler L., Grasl-Kraupp B., Hogstrand C. (2017). Appropriateness to set a group health based guidance value for nivalenol and its modified forms. EFSA J..

[176] IARC Monographs Priorities Group (2019). Advisory Group recommendations on priorities for the IARC monographs. Lancet Oncol..

[177] Forner A., Llovet J., Bruix J. (2015). Diet, Nutrition, Physical Activity and Liver Cancer. https://www.wcrf.org/sites/default/files/Liver-cancer-report.pdf.

[178] Knutsen H.K., Alexander J., Barregård L., Bignami M., Brüschweiler B., Ceccatelli S., Cottrill B., Dinovi M., Edler L., Grasl-Kraupp B. (2018). Effect on public health of a possible increase of the maximum level for ‘aflatoxin total’ from 4 to 10 μg/kg in peanuts and processed products thereof, intended for direct human consumption or use as an ingredient in foodstuffs. EFSA J..

[179] Bbosa G.S., Kitya D., Lubega A., Ogwal-Okeng J., Anokbonggo W.W., Kyegombe D.B. (2013). Review of the biological and health effects of aflatoxins on body organs and body systems. Aflatoxins-Recent Adv. Future Prospect..

[180] FAO/WHO Expert Committee on Food Additives (2017). Evaluation of Certain Contaminants in Food. http://www.who.int/bookorders.

[181] Huang M.N., Yu W., Teoh W.W., Ardin M., Jusakul A., Ng A.W.T., Boot A., Abedi-Ardekani B., Villar S., Myint S.S. (2017). Genome-scale mutational signatures of aflatoxin in cells, mice, and human tumors. Genome Res..

[182] McCullough A.K., Lloyd R.S. (2019). Mechanisms underlying aflatoxin-associated mutagenesis—Implications in carcinogenesis. DNA Repair.

[183] Turner P.C., Sylla A., Diallo M.S., Castegnaro J.-J., Hall A.J., Wild C.P. (2002). The role of aflatoxins and hepatitis viruses in the etiopathogenesis of hepatocellular carcinoma: A basis for primary prevention in Guinea-Conakry, West Africa. J. Gastroenterol. Hepatol..

[184] Wild C.P., Miller J.D., Groopman J.D., IARC (2015). Mycotoxin control in low- and middle-income countries. Working Group Report.

[185] Rheeder J.P., Marasas W.F., Vismer H.F. (2002). Production of fumonisin analogs by Fusarium species. Appl. Environ. Microbiol..

[186] Kouadio J.H., Mobio T.A., Baudrimont I., Moukha S., Dano S.D., Creppy E.E. (2005). Comparative study of cytotoxicity and oxidative stress induced by deoxynivalenol, zearalenone or fumonisin B1 in human intestinal cell line Caco-2. Toxicology.

[187] Howard P.C., Eppley R.M., Stack M.E., Warbritton A., Voss K.A., LorentZEA R.J., Kovach R.M., Bucci T.J. (2001). Fumonisin b1 carcinogenicity in a two-year feeding study using F344 rats and B6C3F1 mice. Environ. Health Perspect..

[188] Riley R.T., Torres O., Matute J., Gregory S.G., Ashley-Koch A.E., Showker J.L., Mitchell T., Voss K.A., Maddox J.R., Waes J.B.G. (2015). Evidence for fumonisin inhibition of ceramide synthase in humans consuming maize- based foods and living in high exposure communities in Guatemala. Mol. Nutr. Food Res..

[189] Gardner N.M., Riley R.T., Showker J.L., Voss K.A., Sachs A.J., Maddox J.R., Gelineau-van Waes J.B. (2016). Elevated nuclear sphingoid base-1-phosphates and decreased histone deacetylase activity after fumonisin B1 treatment in mouse embryonic fibroblasts. Toxicol. Appl. Pharmacol..

[190] Persson E.C., Sewram V., Evans A.A., London W.T., Volkwyn Y., Shen Y.-J., van Zyl J.A., Cheng G., Lin W., Shephard G.S. (2012). Fumonisin B1 and risk of hepatocellular carcinoma in two Chinese cohorts. Food Chem. Toxicol..

[191] Shephard G.S., Van Der WesthuiZEA L., Sewram V. (2007). Biomarkers of exposure to fumonisin mycotoxins: A review. Food Addit. Contam..

[192] Vidal A., Claeys L., Mengelers M., Vanhoorne V., Vervaet C., Huybrechts B., De Saeger S., De Boevre M. (2018). Humans significantly metabolize and excrete the mycotoxin deoxynivalenol and its modified form deoxynivalenol-3-glucoside within 24 hours. Sci. Rep..

[193] Frangiamone M., Cimbalo A., Alonso-Garrido M., Vila-Donat P., Manyes L. (2021). *In vitro* and *in vivo* evaluation of AFB1 and OTA-toxicity through immunofluorescence and flow cytometry techniques: A systematic review. Food Chem. Toxicol..

[194] Jarvis B.B., Miller J.D. (2005). Mycotoxins as harmful indoor air contaminants. Appl. Microbiol. Biotechnol..

[195] Serra R., Braga A., Venâncio A. (2005). Mycotoxin-producing and other fungi isolated from grapes for wine production, with particular emphasis on Ochratoxin A. Res Microbiol..

[196] Chinaza G.A., Erick N.O., Hannington T., Victory S.I., Ikechukwu O.A. (2021). Aflatoxin B1 Production, Toxicity, Mechanism of Carcinogenicity, Risk Management, and Regulations. Arch. Anim. Poult. Sci..

[197] He X.Y., Tang L., Wang S.L., Cai Q.S., Wang J.S., Hong J.Y. (2006). Efficient activation of aflatoxin B1 by cytochrome P450 2A13, an enzyme predominantly expressed in human respiratory tract. Int. Cancer.

[198] Eaton D.L., Groopman J.D. (2013). Toxicology of Aflatoxins: Human Health, Veterinary, and Agricultural Significance.

[199] Yates M.S., Kwak M.-K., Egner P.A., Groopman J.D., Bodreddigari S., Sutter T.R., Baumgartner K.J., Roebuck B.D., Liby K.T., Yore M.M. (2006). Potent protection against aflatoxin-induced tumorigenesis through induction of Nrf2-regulated pathways by the triterpenoid 1-[2-cyano-3-,12-dioxooleana-1,9(11)-dien-28-oyl] imidazole. Cancer Res..

[200] Wichmann G., Herbarth O., Lehmann I. (2002). The mycotoxins citrinin, gliotoxin, and patulin affect interferon-γ rather than interleukin-4 production in human blood cells. Environ. Toxicol.

[201] Dönmez-Altuntas H., Dumlupinar G., Imamoglu N., Hamurcu Z., Liman B.C. (2007). Effects of the mycotoxin citrinin on micro- nucleus formation in a cytokinesis-block genotoxicity assay in cultured human lymphocytes. J. Appl. Toxicol..

[202] Flajs D., Peraica M. (2009). Toxicological properties of citrinin. Arh. Hig. Rada Toksikol..

[203] Chu Y.-M., Jeon J.-J., Yea S.-J., Kim Y.-H., Yun S.-H., Lee Y.-W., Kim K.H. (2002). Double-stranded RNA mycovirus from Fusarium graminearum. Appl. Environ. Microbiol..

[204] Gelderblom WC A., Smuts C.M., Abel S., Snyman S.D., Cawood M.E., Van der Westhuizen L., Swanevelder S. (1996). Effect of fumonisin B1 on protein and lipid synthesis in primary rat hepatocytes. Food Chem. Toxicol..

[205] Missmer S., Hendricks K., Suarez L., Larsen R., Rothman I. (2000). Fumonisins and neural tube defects. Epidemiology.

[206] Awuchi C.G., Ondari E.N., Ofoedu C.E., Chacha J.S., Rasaq W.A., Morya S., Okpala C.O.R. (2021). Grain Processing Methods’ Effectiveness to Eliminate Mycotoxins: An Overview. Asian J. Chem..

[207] Rocha O., Ansari K., Doohan F.M. (2005). Effects of trichothecene mycotoxins on eukaryotic cells: A review. Food Addit. Contam..

[208] Diamond M., Reape T.J., Rocha O., Doyle S.M., Kacprzyk J., Doohan F.M., McCabe P.F. (2013). The fusarium mycotoxin deoxynivalenol can inhibit plant apoptosis-like programmed cell death. PLoS ONE.

[209] Desmond O.J., Manners J.M., Stephens A.E., Maclean D.J., Schenk P.M., Gardiner D.M., Munn A.L., Kazan K. (2008). The Fusarium mycotoxin deoxynivalenol elicits hydrogen peroxide production, programmed cell death and defence responses in wheat. Mol. Plant Pathol..

